# Activation of p53-regulated pro-apoptotic signaling pathways in PrP-mediated myopathy

**DOI:** 10.1186/1471-2164-10-201

**Published:** 2009-04-28

**Authors:** Jingjing Liang, Debra Parchaliuk, Sarah Medina, Garrett Sorensen, Laura Landry, Shenghai Huang, Meiling Wang, Qingzhong Kong, Stephanie A Booth

**Affiliations:** 1 Department of Pathology, Case Western Reserve University, Cleveland, OH 44106, USA; 2 Molecular PathoBiology, National Microbiology Laboratory, Winnipeg, Manitoba, R3E 3R2, Canada; 3 Prion Diseases Program, National Microbiology Laboratory, Winnipeg, Manitoba, R3E 3R2, Canada; 4 Department of Medical Microbiology and Infectious Diseases, Faculty of Medicine, University of Manitoba, Winnipeg, MB, R3E 0W3, Canada

## Abstract

**Background:**

We have reported that doxycycline-induced over-expression of wild type prion protein (PrP) in skeletal muscles of Tg(HQK) mice is sufficient to cause a primary myopathy with no signs of peripheral neuropathy. The preferential accumulation of the truncated PrP C1 fragment was closely correlated with these myopathic changes. In this study we use gene expression profiling to explore the temporal program of molecular changes underlying the PrP-mediated myopathy.

**Results:**

We used DNA microarrays, and confirmatory real-time PCR and Western blot analysis to demonstrate deregulation of a large number of genes in the course of the progressive myopathy in the skeletal muscles of doxycycline-treated Tg(HQK) mice. These include the down-regulation of genes coding for the myofibrillar proteins and transcription factor MEF2c, and up-regulation of genes for lysosomal proteins that is concomitant with increased lysosomal activity in the skeletal muscles. Significantly, there was prominent up-regulation of p53 and p53-regulated genes involved in cell cycle arrest and promotion of apoptosis that paralleled the initiation and progression of the muscle pathology.

**Conclusion:**

The data provides the first *in vivo *evidence that directly links p53 to a wild type PrP-mediated disease. It is evident that several mechanistic features contribute to the myopathy observed in PrP over-expressing mice and that p53-related apoptotic pathways appear to play a major role.

## Background

Cellular prion protein (PrP^C^) is a ubiquitous glycosylphosphatidyl-inositol (GPI) anchored glycoprotein that has gained enormous attention as the central factor in prion diseases [1]. In these diseases PrP^C ^is converted through conformational change to a pathological form (PrP^Sc^) that self-replicates using PrP^C ^as the substrate. The normal functions of PrP^C ^remain elusive despite concerted efforts. PrP^C ^has been implicated in CNS development, neurite outgrowth and neuronal survival, early synaptic neuronal transmission and reorganization of neuronal circuitry within the hippocampus, regulation of circadian rhythm, memory formation and cognition, maintenance of Ca^2+^-activated K^+ ^currents of hippocampal CA1 pyramidal neurons, protection against brain injury in rat and mouse models of ischemic stroke, and in T cell development and function [2]. Over-expression of PrP^C ^has been shown to exert a protective effect in BAX and TNFα-mediated cell death and conversely a pro-apoptotic function in studies of staurosporine-induced cell death [3-5]. It has also been demonstrated that depletion of endogenous PrP reduces susceptibility to staurosporine-induced caspase 3 and p53 activation [6].

In a previous study we generated transgenic mice, Tg(HQK), that express human PrP^C ^exclusively in the skeletal muscles under tight regulation by doxycycline [7]. We found that induced over-expression of PrP^C ^in the muscles leads to a progressive primary myopathy characterized by increased variation of myofiber size, centrally located nuclei and endomysial fibrosis, in the absence of cytoplasmic inclusions, rimmed vacuoles, or any evidence of a neurogenic disorder [7]. While the pathogenic mechanism of the PrP-mediated myopathy was not determined, an interesting observation was that the myopathy was accompanied by preferential accumulation of an N-terminal-truncated PrP^C ^fragment, which was confirmed to be the C1 fragment [7] resulting from normal PrP^C ^processing [8-12]. The C1 fragment is also found in the skeletal muscles of wild-type mouse, but at a much lower level and a molar ratio of close to 1:1 over full-length PrP^C^, in contrast to a ratio of 3:1 in the Dox-induced Tg(HQK) model [7].

A number of studies have shown the expression of N-terminus truncated forms of PrP^C ^to be associated with toxicity in animal models [13,14]. The protein Doppel, which is homologous to the C-terminus of PrP, has also been shown to be cytotoxic when ectopically expressed in neurons [15-17]. In both cases, the toxicity can be abrogated by the co-expression of full length PrP^C ^[18,19]. The C1 fragment has also been reported to potentiate staurosporine-induced toxicity via caspase 3 activation in cultured cells [20], but this toxic effect is similar to what was reported for full-length PrP^C ^[5,21,22]. We hypothesize that the high levels of the C1 fragment that accumulate in Dox-treated Tg(HQK) mice is largely responsible for the toxic effect that leads to the development of myopathy in these mice. In order to understand the molecular mechanism that underlies this PrP toxicity, we have performed microarray analysis to determine gene regulatory networks that are triggered following overexpression of PrP^C ^in the skeletal muscles of Tg(HQK) mice.

## Methods

### Animals and Treatment

The doxycycline-inducible Tg(HQK) mice were described previously [7]. The HQK transgene contained two genes: reverse tetracycline responsive transcription activator (rtTA) under the control of the mouse PrP promoter of the half genomic PrP clone, and human PrP ORF regulated by the tetracycline-responsive promoter (tetO-hCMV*-1) from the core plasmid [23]. The Tg(HQK) mice were generated in the FVB background, and Tg(HQK)/*Prnp*^*o*/*o *^mice were obtained through breeding with the Zurich I PrP-null mice [24] in FVB background. Line Tg(HQK)18, referred to as Tg(HQK) for simplicity, was used for this study.

### Animal Treatment and Specimen Collection

Wild type (WT), PrP-null (KO), and Tg(HQK) mice were fed food pellets either lacking or containing 6 g doxycycline (Dox)/kg food (Bio-Serv) to induce PrP^C ^expression. Skeletal muscles from the quadriceps of hind legs were removed at day 0, 4, 7, 14, 30 and 60 days following administration of Dox. For immunoblot and microarray analysis, the muscle tissues were immediately frozen on dry ice, and stored at -80°C.

### RNA Isolation

Total RNA was isolated from frozen skeletal muscle using the RNeasy skeletal muscle RNA isolation kit (Qiagen) following the manufacturer's specifications. The total RNA preparations were further treated with Turbo DNA-Free DNase (Ambion) to remove residual genomic DNA contamination, and examined with a Bioanalyzer 2100 (Agilent) for purity and quantity.

### RNA Amplification and Labeling for Microarray Analysis

Total RNA was amplified and labeled for microarray analysis using the AminoAllyl Message Amp II aRNA amplification kit (Ambion) following the manufacturer's specifications. In brief, 1 μg total RNA was reverse transcribed to first-strand cDNA, followed by subsequent second-strand cDNA synthesis. In vitro transcription to synthesize amplified aRNA was performed and the resultant aRNA quantified. Ten to fifteen micrograms of aRNA was designated as reference (WT) or experimental (KO, HQK), and then coupled to either Alexa Fluor succinimidyl ester 555 or Alexa Fluor succinimidyl ester 647 dye in 30% DMSO/coupling buffer in the dark at room temperature for 1 hour. Each sample was labeled individually with both Alexa Fluor 555 and 647 for subsequent dye-swapped hybridizations to account for intensity bias. Uncoupled dyes were removed and labeled aRNA purified following the manufacturer's specifications.

### cDNA Microarrays

A total of 16,315 cDNA expressed sequence tags from the Brain Molecular Anatomy Project (BMAP) mouse brain library  were spotted in duplicate onto CMT-GAPS Gamma Amino Propyl Silane coated glass slides (Corning) using the Virtek Chip Writer. Five micrograms of both reference (WT) and experimental (KO and HQK) Alexa Flour labeled aRNA were used in each competitive hybridization. Each labeled aRNA was resuspended in 35 μl DIG Easy Hyb™ hybridization buffer (Roche) containing 20 μg mouse cot1 DNA and 20 μg poly(A)-DNA to block non-specific hybridization. Three biological replicate samples from each of the reference and experimental groups were combined, heated for 5 minutes at 95°C, then cooled and maintained at 42°C. The labeled aRNA sample mixtures were added to a BMAP microarray and incubated in the dark at 42°C overnight to competitively hybridize to reference and experimental samples. The number of slides hybridized in each experiment corresponded to the number of biological replicates in each group of experimental interest. Following hybridization, the slides were washed once in low stringency wash buffer (1× SSC, 0.2% SDS) preheated to 42°C for 5 minutes, once in high stringency wash buffer (0.1× SSC, and 0.2%SDS) for 5 minutes at room temperature, and then once in 0.1× SSC for 5 minutes at room temperature. The slides were analyzed in two channels using the Agilent HT microarray scanner (Agilent). Raw, background and net intensity values were collected using Array-Pro software (Media Cybernetics). In order to account for variation in fluorescence, LOWESS sub-grid normalization was performed by Gene Traffic software (Iobion), and the subsequent normalized log2 ratios obtained. The resulting ratio between reference and experimental signals for each individual gene was used as a measure of differential gene expression using EDGE (Extraction of Differential Gene Expression), an open source software program for the significance analysis of DNA microarray experiments .

EDGE implements statistical methodology specifically designed for time course experiments [25]. A significance measure is assigned to each gene via the Q value (false discovery rate) methodology [26]. We selected a Q-value cutoff to display the genes that met our significance threshold. We performed a "between class" analysis of the data over time; the class variables, or biological groups, were the PrP over-expressing mice [Tg(HQK)] and the PrP-KO mice.

### Agilent Whole Mouse Genome Oligonucleotide Microarrays

One microgram of each Alexa Fluor 555 and 647 labeled samples as prepared above were fragmented, reference and experimental samples together, in 250 μl fragmentation mix in preparation for hybridization to Agilent's Whole Mouse Genome 44 K oligonucleotide microarrays. Following the manufacturer's protocol, an equal volume of 2× hybridization buffer was added to stop aRNA fragmentation and prepare the samples for hybridization. Four hundred fifty microliters of each mixture containing the reference and experimental samples was then added to an individual slide hybridization assembly and allowed to rotate at 4 rpm at 65°C for 17 hours. Slides were washed and scanned as recommended in the protocol, then analyzed using Agilent Feature Extraction Software. Raw, background and net intensity values were collected. A linear and LOWESS normalization correction method was selected in order to account for variations in fluorescence. A two-sided t-test of feature versus background, set at a p value of 0.05, was used to obtain a list of genes whose log_10 _ratios were significant.

### Validation of Gene Expression Using Quantitative PCR

Some of the genes that appeared to be differentially regulated were confirmed with quantitative real-time PCR (qRT-PCR), using probe specific TaqMan gene expression assays on the Applied Biosystems 7500 Fast Real-Time PCR System. 100 ng of total RNA previously isolated and used for microarray analysis was reverse transcribed using the High Capacity cDNA Reverse Transcription kit. Subsequently, 1 μl from each reverse transcription reaction was assayed in a 20 μl single-plex reaction for real-time quantification with the 7500 Fast PCR System using probes specific to those genes of interest. Each sample was run in biological triplicate, of which 3 technical replicates were performed. GAPDH was used as the endogenous control, and gene expression of target genes for KO and HQK samples were quantitatively measured relative to the WT samples. Relative quantification values were determined using the 2^-ΔΔct ^method, and expressed as fold-change over WT.

### Immunoblot Analysis

Mouse skeletal muscle tissues were homogenized in lysis buffer containing 50 mM Tris (pH 7.5), 200 mM sodium chloride, 0.5% sodium deoxicholate, and 5 mM EDTA. Protein concentrations were determined by the BCA protein assay (Pierce). After addition of LDS sample buffer (Invitrogen) and sample reducing agents (Invitrogen), the homogenates were denatured at 100°C for 10 minutes, and the proteins were resolved on 10% NuPage Tris-Bis Gels (Invitrogen) and blotted onto nitrocellulose membranes (Invitrogen). For p53 protein detection, the membrane was incubated with a monoclonal anti-p53 antibody that detects total p53 proteins (Cell Signaling) (1:2000 diluted in 5% milk, 1× TBS, 0.1% Tween-20) at 4°C with gentle shaking overnight. For MEF2C detection, the membrane was incubated with a rabbit polyclonal anti-MEF2C antibody (Cell Signaling) (1:5000 diluted in 0.5% normal goat serum [Vector Laboratories], 1× TBS, 0.1% Tween-20) at 4°C with gentle shaking overnight. The blots were developed with the Immobilon Western Chemiluminescent HRP substrate (Millipore) according to the manufacturer's instructions. Skeletal muscle actin was probed with a rabbit polyclonal antibody (Abcam) (1:5000 diluted in 0.5% normal goat serum, 1× TBS, 0.1% Tween-20) similarly after stripping the blots with a stripping buffer containing 1.4% 2-mercaptoethanol, 2% SDS and 62.5 mM Tris (pH 6.8). The western blots were scanned and the protein bands were quantified with the UN-SCAN-IT gel 6.1 software (Silk Scientific).

### Accession numbers

The BMAP and Agilent microarray related data were submitted to Gene Expression Omnibus (GEO) under accession number: [GSE12576]

## Results

### Induction of PrP^C ^Specifically in the Skeletal Muscle of Transgenic Mice Results in a Temporally Regulated Transcriptional Profile

**Figure 1:**
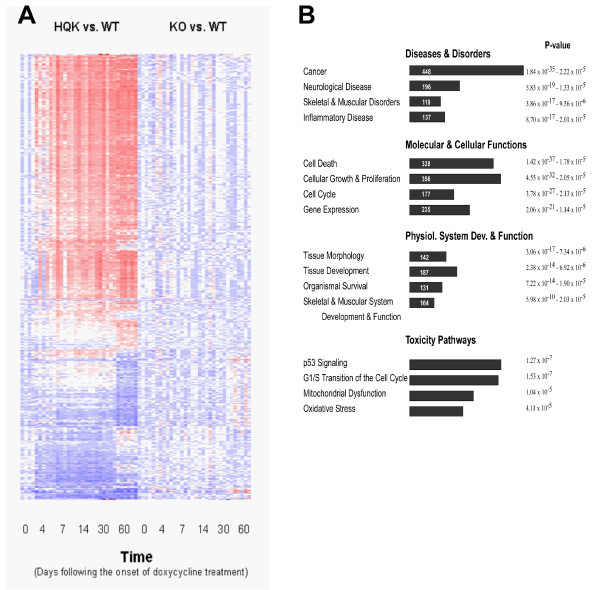
**Clustering of gene expression data**. **A**. Measurements of relative gene expression for 6 time points (after 0, 4, 7, 14, 30, 60 days of Dox treatment) in Tg(HQK) mice (HQK) and PrP knockout mice (KO). Mice were treated with 6 g Dox/kg food, and three animals were taken at each time point as indicated. Total RNA was extracted from skeletal muscles (quadriceps) from the hind legs and subjected to microarray analysis, yielding expression profiles of genes with normalized expression ratios. Red and purple represent relative over-expression and under-expression, respectively, and the color intensity represents the magnitude of digression. **B**. Bioinformatic analysis (Ingenuity Pathways Analysis) to determine the top biological functions and associated p values of the selected genes is shown. The top four categories are listed for diseases and disorders, molecular and cellular functions, physiological system and development and function, and pathways associated with toxicity.

The transgenic mice [Tg(HQK)] used in this study have been described previously, in which PrP^C ^is exclusively expressed in skeletal muscles under the strict control of doxycycline (Dox) and the induced over-expression of PrP^C ^leads to a progressive primary myopathy [7]. To determine the temporal patterns of gene expression that accompany the induced myopathy, we carried out microarray analysis of skeletal muscles from Tg(HQK) mice, wild-type FVB mice (WT) and PrP-knockout control mice (KO) using a 16,315-gene cDNA array constructed in our laboratory. Skeletal muscles from the hind legs (quadriceps) of the mice were collected at 0, 4, 7, 14, 30, and 60 days following administration of Dox. Three animals were taken at each time point for each of the three mouse lines [Tg(HQK), WT, KO]. Temporally regulated genes in the quadriceps of Tg(HQK) and KO, in comparison to WT, were identified using EDGE (extraction and analysis of differential gene expression), a significance method for analyzing time course microarray data [27,28]. A Q value cut-off of 0.05, and a fold change of 3 for at least one time-point, was the criteria used for the selection of differentially expressed genes. In the muscles of Dox-treated Tg(HQK) mice, 1499 differentially expressed genes were identified in comparison with similarly treated, age-matched WT mice; a cluster plot of all differentially expressed genes based on similarities in their expression profiles is shown in Figure 1A. In contrast, only 13 genes showed significant differential expression in the muscles of KO mice in comparison with similarly treated, age-matched WT mice. To verify the expression of genes identified on our cDNA array, and to sample a more complete set of genes covering the whole mouse genome, we purchased additional microarrays from Agilent technologies. These arrays consisted of 44,000 oligonucleotide probes representing the whole mouse genome. We re-examined the day 14 samples since the majority of the 1499 temporally deregulated genes showed differential expression at this time point. A two-sided t-test of feature versus background, set at a p value of 0.05, was used to obtain a list of genes whose log_10 _ratios were significant. This list was in good agreement with the data from our in-house manufactured cDNA array, confirming the deregulation of almost two-thirds of genes originally identified by the cDNA array, in addition a set of genes which were not represented by probes on our in-house cDNA arrays were identified. In total, 1265 selected genes were annotated in the Ingenuity Pathway Analysis (IPA) database and are provided as Additional file 1 (up-regulated) and Additional file 2 (down-regulated). A summary of the most common biological functions and toxicity-related pathways associated with these genes is shown in Figure 1B. Gene ontology analysis revealed that up-regulated genes were particularly enriched for genes involved in development, cell cycle regulation, programmed cell death, lipid metabolism and ion homeostasis (Table 1). Down-regulated gene ontology categories were enriched for genes involved in cellular energy metabolism, particularly carboxylic acid metabolism, protein metabolism and muscle developmental processes (Table 2).

**Table 1:** List of genes belonging to some of the most significantly up-regulated Gene Ontology Categories

**Description**	**Gene Name**
cell development	ARF6, KIF5C, PRM2, NEB, SOX9, NDN, RUNX1, FCER1G, ENAH, PRKDC, GADD45G, PURB, METRN, BIRC5, TRADD, LGALS1, EPHB1, TRIM35, GPX1, STMN3, E2F2, NEFL, DEDD, RHOA, JMY, MAL, DCX, CASP14, UNC5B, BNIP1, CD28, GDNF, ITGB1BP3, ALS2CR2, NFKB1, TIMP1, CARD10, SEMA6A, DAB1, CHRNA1, UCHL1, TNFRSF12A, HSPA1A, MYOG, AKT1S1, PIP5K1C, TRIAP1, PMAIP1, MT3, SOCS2, GADD45B, ABI2, TNNT2, GSK3B, SGPP1, RPS6KB1, HIPK2, IGFBP3, PERP, PPP1R13B, CDK5R1, HOOK1, EDA2R, CTF1, EHMT2, ITGA3, SOX10, HIPK3, E2F1, BCL2L13, PURA, YBX2, IBRDC2, APP, BOK, TNP1, FAF1, PHLDA1, CAMK1D, CSPG4, DOCK1, FARP2, DIABLO, GDF11, ZFP91, PEG3, PTPRC, BAK1, RHOT1, NRAS, CDKN1A, NAB2, DAP3

cell cycle	ANLN, CDC27, PRM2, TIMELESS, RB1, TACC3, SMARCB1, GADD45G, CDC14A, BIRC5, INCENP, CHES1, UHRF2, PDGFB, CGREF1, MIS12, E2F2, CDK6, PSMD2, JMY, CITED2, SUV39H2, PPP2R3A, CD28, ALS2CR2, PLK2, MERTK, CLASP1, CRKL, PRC1, TRIAP1, GADD45B, CPEB1, FOS, GSK3B, HIPK2, EHMT2, SPAG5, RANBP1, E2F8, E2F1, PLEKHO1, GAK, CCRK, PURA, HDAC5, RASSF4, APP, DHX16, E2F3, THPO, MKI67, BIN1, PTPRC, RGS2, ABL1, ANAPC1, NRAS, CDKN1A, JUNB, MDM2, ITGAE

programmed cell death	ARF6, SOX9, FCER1G, PRKDC, PURB, GADD45G, BIRC5, TRADD, TRIM35, GPX1, E2F2, NEFL, RHOA, DEDD, JMY, MAL, CASP14, GDNF, BNIP1, CD28, UNC5B, ALS2CR2, NFKB1, CARD10, SEMA6A, TNFRSF12A, HSPA1A, AKT1S1, PMAIP1, TRIAP1, GADD45B, SGPP1, GSK3B, IGFBP3, HIPK2, PERP, CDK5R1, PPP1R13B, EDA2R, HIPK3, E2F1, BCL2L13, PURA, IBRDC2, APP, BOK, FAF1, PHLDA1, CAMK1D, DOCK1, DIABLO, ZFP91, PEG3, PTPRC, BAK1, RHOT1, NRAS, CDKN1A, DAP3

cellular lipid metabolic process	ISYNA1, PRKAA1, LCAT, NEB, SULT2B1, PIP5K1C, B4GALNT1, VLDLR, FDPS, SERINC2, SGPP1, LDLR, ADIPOR2, RDH11, SYK, CDS2, SNCA, PRKAG2, MYO5A, ELOVL6, HEXB, CDS1, CD81, BMPR1B, ST6GALNAC6, SOAT1, FADS3, PIP5K1A, CHKB, PIGO, ELOVL3, UGCG, AYTL2, SLC37A4, AGPAT3, PBX1, AGPAT2, SYNJ1, INSIG1, PIGK, HMGCS2, PRKAB2, ACBD3, CYB5R3, PISD, SERINC1, MTMR7, HEXA

cellular ion homeostasis	CHRNA1, APP, ATOX1, CHRNG, APLP2, SV2A, CHRNB4, MT3, NDN, RYR3, PRND, ATP2A2, PTPRC, BAK1, SLC37A4, MT4, SYPL2, ANXA7, PRNP, SLC39A5, HEXB

**Table 2:** List of genes belonging to some of the most significantly down-regulated Gene Ontology Categories

**Description**	**Gene Name**
carboxylic acid metabolic process	NR3C1, TARSL2, AHCY, SHMT2, PTGES3, LYPLA1, IRG1, MCCC1, PRKAG1, MAT2B, CYP39A1, MCFD2, IDH2, GLUL, IARS, PYCR2, FBP2, PLP1, ABAT, ADIPOR1, LYPLA2, BCKDHA, CROT, GPD2, CAV1, MTHFD1, SH3GLB1, ACADSB

protein metabolic process	PPP3CB, BZW2, PPP2CB, AGA, CDC16, CHEK2, HERC2, UBE2B, PRMT7, NCKAP1, EIF4A2, CCT2, TLK2, KLK8, PDPK1, CSE1L, OMA1, UBQLN2, SLC30A9, PRKAR2A, HAT1, CAPZA2, CLK1, CPA3, LCK, CAMK2G, CAV1, MTM1, PSMB9, HUWE1, UBE2D1, PRKRIR, FKBP8, FKBP4, ARAF, RPS6KC1, PPP2R2A, VWF, CCT6A, GART, EPHA7, EIF3S6, WWP1, DVL1, IARS, ASPH, HTRA2, RNF6, RNF8, UBE2A, MCPT4, HS3ST5, CUL3, PCTK3, EEF2, UBE2G1, MMP13, UQCRC2, PRPF4B, AP3M1, BRCC3, SH3GLB1, RPL36, TARSL2, CDKL2, CAMK1, USP15, ULK2, BACE2, HECTD1, DNAJC12, ITGB4BP, CRY1, RAD21, FBXL5, DMD, MGRN1, RCHY1, IPO11, VBP1, USP1, VPS35, YME1L1, RPL22, COPB1, LGTN, GLMN, RSL1D1, RPL4, SUV420H2, ETF1, MAP2K5, USP38, EGLN1, TBCE, CUZD1, FURIN, PAIP1, CDC25B, EIF4G2, IFNAR1, TRIM23, CAV2, PSMD6, PIGY, LAP3

muscle development	CSRP3, DMD, MYL2, TSC1, MYH7, CAV2, MTM1, CAV1, ACTC1, DVL1, ACTG2, MYH6, CACNA1S, MEF2C

### PrP^C ^Over-expression Regulates Multiple Targets with Established Roles in Myopathy

**Figure 2:**
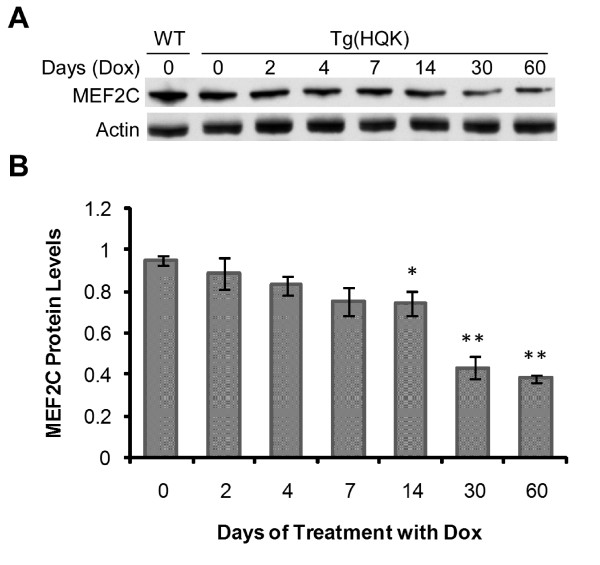
**MEF2C protein level is down-regulated in the skeletal muscles of Tg(HQK) mice treated with doxycycline (Dox)**. Tg(HQK) mice were treated with 6 g Dox/kg food for 0–60 days as indicated, and three animals were taken at each time point. Skeletal muscles (quadriceps) from the hind legs were subjected to immunoblot analysis in three blots. Fifteen micrograms of total proteins was loaded for each sample. Skeletal muscle (quadriceps) sample from an untreated wild type FVB mouse (WT) serves as the control to normalize data from the triplicate blots. **A**. A representative immunoblot probed with anti-MEF2C antibody followed by probing with an anti-actin antibody after stripping. **B**. Plot of the MEF2C protein levels over increasing duration of Dox treatment. The MEF2C protein level for each sample was normalized against the actin level in each blot and expressed as the ratio against the normalized MEF2C protein level in the untreated wild type FVB mouse on the same blot. The error bars denote standard errors calculated from the three blots. The bars with asterisk(s) indicate a statistically significant difference when compared to the 0 day Tg(HQK) samples. *p < 0.05; ** p < 0.001.

Many of the gene expression changes identified in the Tg(HQK) muscle are consistent with the observed progressive atrophy, which is characterized by a decrease in myofiber size and total muscle mass accompanied by a concomitant accumulation of lysosomes. Specific changes included a significant down-regulation of genes coding for the myofibrillar proteins MYH2, MYH6, MYH7, MYL2, MYL3, and an increase in expression of the transcription regulator MDFI (MyoD Family Inhibitor) that acts as a negative regulator of myogenic proteins, and induction of MyoG (myogenin), a muscle-specific transcription factor that can induce myogenesis in a variety of cell types in tissue culture. The MEF2C (Myocyte Enhancer Factor 2C) gene was also down-regulated in Dox-induced Tg(HQK) muscles. Immunoblot analysis showed that there was statistically significant reduction of MEF2C protein level in the skeletal muscle from day 14 of Dox treatment, and the reduction reached 50% after 30 days of Dox treatment (Figure 2). MEF2C has been studied extensively in muscle cells. It is a key regulatory transcriptional factor that plays an essential role in the transcriptional control of muscle development as well as remodeling of adult muscles in response to physiologic and pathologic signals [29,30]. It has been reported that MEF2C directly activates the expression of a muscle specific protein kinase Srpk3 and Srpk3-null mice exhibit widespread centronuclear myopathy via an unknown mechanism [31]. We speculate that the down-regulated MEF2C gene expression might play a role in the progressive central nucleus localization observed in the skeletal muscles of Dox-treated Tg(HQK) mice [7] through a reduction of the Srpk3 activity.

A number of lysosomal peptidases were up-regulated including CTSS, CTSD, CTSZ, and DPEP2, coincident with an observed accumulation of lysosomes in Tg(HQK) mice over-expressing PrP^C ^[7]. The gene CTSL, which codes for a lysosomal cysteine proteinase, is commonly used as a universal marker for muscle atrophy but was not represented on our arrays [32]. qRT-PCR revealed expression of this gene was induced transiently following PrP^C ^induction, peaking at 7 days following the onset of Dox treatment and returning to the baseline by 60 days post-induction. The genes encoding lysosomal proteins HEXA, HEXB and LAMP1 were also up-regulated at late time points.

Previous studies have shown that the development of muscle atrophy in a number of models of systemic wasting states (fasting, cancer cachexia, renal failure, diabetes) and in disuse atrophy induced by denervation or spinal cord isolation follows a common program of transcriptional changes [33,34]. One of the main features of this program is a general increase in expression of genes involved in proteolysis including both lysosomal proteases, and an ATP-dependent process requiring ubiquitin and the proteasome. The degradation of PrP^C ^and PrP^Sc ^is also believed to involve the proteasome [35], and compromised/inhibited proteasome activity was proposed to lead to accumulation of cytosolic PrP^C ^that is neurotoxic [35]; but the latter notion has been challenged [36,37]. Following induction of PrP^C ^we observed that the expression levels of genes involved in proteasomal protein degradation were for the most part unchanged. Out of the 44 unique proteasome related genes represented on the microarrays, only three (PSMD2, PSMD4, PSMD7) were up-regulated and four (PSMD6, PSMD12, PSMD13 and PSMD14) were down-regulated.

**Figure 3:**
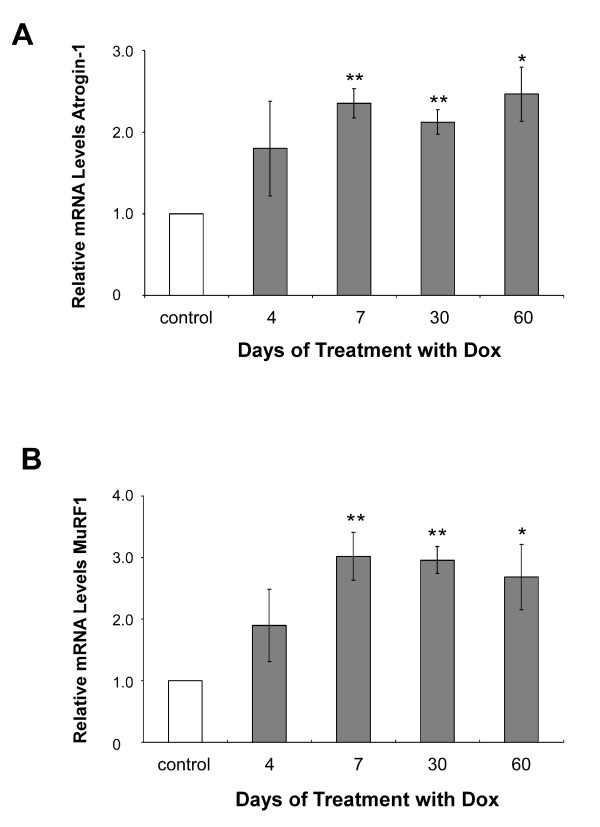
**Real-time PCR analysis of Atrogin-1 and MuRF1**. qRT-PCR analysis of Atrogin-1 (**A**) and MuRF1 (**B**) gene expression in RNA samples from Tg(HQK) mice relative to similarly treated wild-type control mice. Measurements of relative gene expression for 4 time points (over 4–60 days) in mice following treatment with 6 g Dox/kg food beginning on day 0. Total RNA was extracted from skeletal muscles (quadriceps) from the hind legs and subjected qRT-PCR analysis. Results represent the mean ± s.e.m. of triplicate measurements performed. * p < 0.01; ** p < 0.001.

A further feature reported in a number of different models of diseases resulting in muscle atrophy is the substantial up-regulation of two E3 ubiquitin ligases, atrogin-1/MAFbx (FBXO32) and MuRF1 (TRIM63) [38,39], which are generally induced early during the atrophy process. Upon fasting, the rise in atrogin-1 expression precedes the loss of muscle weight; conversely, deletion of either Atrogin-1 or MuRF1 has been shown to significantly alleviate muscle atrophy [39]. Our microarray data did not reveal a significant increase in Atrogin-1 expression in the Tg(HQK) atrophy model and no probe for MuRF-1 was present on either of our array platforms. qRT-PCR was used to determine the expression levels of these two genes, and a small, less than 3-fold increase in the expression of both MuRF1 and Atrogin-1 was detected following induction of PrP^C ^(Figure 3A and 3B); this is much lower than the 10–40 fold increase generally found in other models of muscle atrophy. In a recent study, the induction of the FOXO1 protein (a key activator of atrophy) as well as the fall in PGC-1 alpha and beta (transcriptional co-repressors of Atrogin expression) were identified in numerous types of muscle wasting [40,41]. A 3-fold decrease of FOXO1 and no change in expression of PGC-1 alpha and beta were detected in Dox-treated Tg(HQK) mice. These data suggest only minor involvement of the ubiquitin-proteasome proteolysis pathway in the observed muscle atrophy and a program of transcriptional changes that is not reminiscent of systemic wasting states.

### Activation of p53-Mediated Signaling Pathways Following PrP^C ^Induction in Skeletal Muscle of Tg(HQK)

The dramatic transcriptional response to PrP^C ^over-expression in the muscles of Tg(HQK) mice lacks key features of the common transcriptional program indicative of several reported forms of muscle atrophy. This includes striking de-regulation of over 400 genes involved in cell death and regulation of the cell cycle, which suggests a toxic effect of the over-expressed PrP. Using the Ingenuity Pathway Analysis (IPA) tool, we identified many pathways invoked in response to PrP^C ^over-expression, among which the p53 signaling pathway scored highly with a p value of 1.27 × 10^-7^. Other molecular pathways that scored significantly were the related G1/S transition of the cell cycle (p = 1.53 × 10^-7^), mitochondrial dysfunction (p = 1.04 × 10^-5^) and oxidative stress response (p = 4.11 × 10^-5^) (Table 3).

**Table 3:** List of genes belonging to some of the most significantly de-regulated pathways that have been implicated in toxicity-associated biological processes as resulted by Ingenuity Pathway Analysis (up-regulated are denoted by bold type, down-regulated by plain type)

**Toxicity-Associated Process**	**P-value**	**Genes**
P53 signaling	1.27 × 10^-7^	**BBC3**, **BIRC5**, C12ORF5, **CDKN1A**, CHEK2, **E2F1**, **GADD45B**, **GADD45G**, **GSK3B**, **HIPK2**, **PERP**, **PIK3R5**, **PMAIP1**, **PPP1R13B**, **PRKDC**, **RB1**, **TP63**, **TP53INP1**

G1/S transition of the cell cycle	1.53 × 10^-7^	**ABL1**, CCNE2, **CDK6**, **CDKN1A**, **E2F1**, **E2F2**, **E2F3**, E2F6, **GSK3B**, **HDAC5**, **RB1**, RBL2, **SIN3A**

Mitochondrial dysfunction	1.04 × 10^-5^	AIFM1, **APP**, BACE2, COX6B2, **CYB5R3**, GPD2, **GSR**, HTRA2, NDUFAF1, NDUFB5, **OGDH**, **PRDX3**, SDHA, SDHB, **SNCA**, SOD2, **UQCRC1**, UQCRC2, UQCRFS1

Oxidative Stress	4.11 × 10^-5^	**FOS**, **GPX1**, **GSR**, **GSTA5**, GSTM2, GSTM1, **NFKB1**, **NFKB2**, **PRDX3**, SOD2, **STAT3**

**Figure 4:**
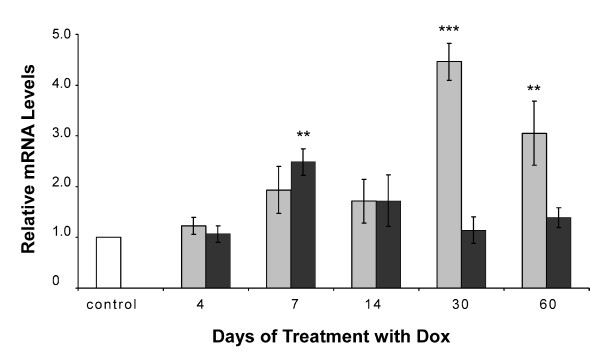
**Real-time PCR analysis of mdm2 and p53**. qRT-PCR analysis of mdm2 (grey) and p53 (black) gene expression in RNA samples from Tg(HQK) PrP over-expressing mice relative to similarly treated wild-type control mice. Measurements of relative gene expression for 5 time points (over 4–60 days) in mice following treatment with 6 g Dox/kg food. Total RNA was extracted from skeletal muscles (quadriceps) from the hind legs and subjected qRT-PCR analysis. Results represent the mean ± s.e.m. of triplicate measurements performed. ** p < 0.01; *** p < 0.001.

**Figure 5:**
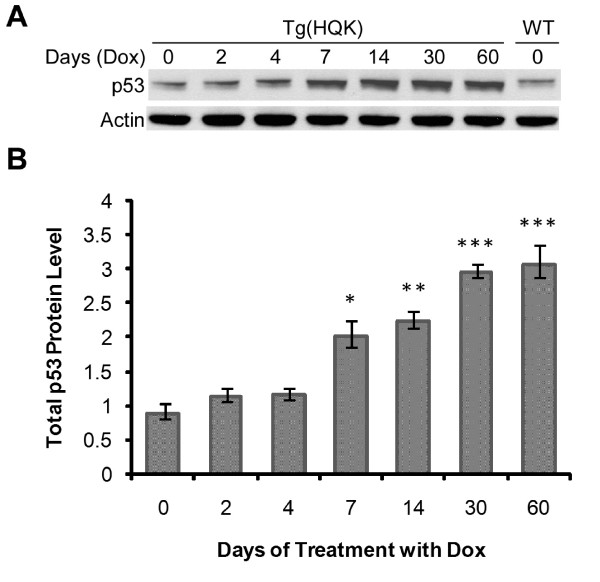
**Total p53 protein level is up-regulated in the skeletal muscles of Tg(HQK) mice treated with doxycycline**. Tg(HQK) mice were treated with 6 g Dox/kg food for 0–60 days as indicated, and three animals were taken at each time point. Skeletal muscles (quadriceps) from the hind legs were subjected to immunoblot analysis in three blots. Twenty micrograms of total proteins was loaded for each sample. Skeletal muscle (quadriceps) sample from an untreated wild type FVB mouse (WT) serves as the control to normalize data from the triplicate blots. **A**. A representative immunoblot probed with anti-p53 antibody followed by probing with an anti-actin antibody after stripping. **B**. Plot of the total p53 protein levels over increasing duration of Dox treatment. The p53 protein level for each sample was normalized against the actin level in each blot and expressed as the ratio against the normalized total p53 protein level in the untreated wild type FVB mouse on the same blot. The error bars denote standard errors calculated from the three blots. The bars with asterisk(s) indicate a statistically significant difference when compared to the 0 day Tg(HQK) samples. *p < 0.05; ** p < 0.01; *** p < 0.001.

The involvement of the p53 signaling pathways was of particular interest as mounting evidence suggests that over-expression of PrP^C ^sensitizes cells to apoptotic stimuli through a p53-dependent pathway [5,20-22]. The p53 gene itself did not meet our selection criteria (a change of 3-fold or more in at least one time point) as significantly deregulated in the microarray analysis; however qRT-PCR showed it to be marginally up-regulated from day 7 following the onset of PrP^C ^induction. This transient over-expression was low, approximately 1.5–2.5 fold, but statistically significant in all Tg(HQK) mice tested (Figure 4). However, regulation of p53 is known to take place mostly at the level of translation [42]. In accordance with this, immunoblot analysis of the levels of total p53 protein in the skeletal muscle (quadriceps) of Tg(HQK) mice, shown in Figure 5A and 5B, revealed a moderate but significant accumulation of p53 protein beginning at day 7 following the commencement of doxycycline treatment and rising to over 3-fold over age-matched WT controls 30–60 days post Dox induction. Activation of p53 is kept in check by its negative regulator MDM2 (mouse double minute 2) in a negative feedback regulatory loop since activated p53 induces expression of MDM2 [42]. We found that the levels of MDM2 were only marginally changed at early time points but were significantly up-regulated at the later time points (30 and 60 days), congruent with the accumulation of p53 protein (Figure 5). The moderate increase in p53 in the muscles of Dox-treated Tg(HQK) mice is consistent with the observed gradual and progressive muscle wasting.

### Deregulation of Genes Involved in p53-Dependent G_1 _Cell Cycle Arrest and Apoptosis

**Figure 6:**
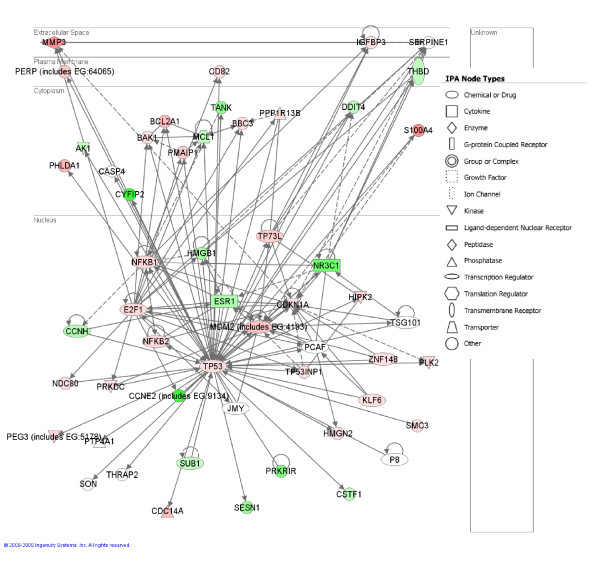
**p53-regulated pathway analysis using the Ingenuity Pathway Knowledge Base (IPKB)**. This figure illustrates potential functional relationships of TP53 responsive genes de-regulated in the muscles of Dox-treated Tg(HQK) mice. Direct (solid lines) and indirect (dashed lines) interactions reported for these genes (grey shading) in the IPKB database. Color shading corresponds to the type of de-regulation: red for up-regulated genes, and green for down-regulated genes. The shape of the node indicates the major function of the protein (see key), and a line denotes binding of the products of the two genes while a line with an arrow denotes 'acts on'.

Systematic examination of the genes differentially expressed following PrP^C ^over expression revealed over 60 genes that were annotated, or cited in PubMed, as being p53 responsive genes. We used the IPA tool to build a network of potential regulatory interactions between the products of these genes; the resulting network is shown in Figure 6. The genes making up this network are primarily involved in the regulation of the cell cycle and cell death. A number of these are transcription factors including the proinflammatory regulator NF-κB which has been shown to be activated in degenerating muscle of Duchenne muscular dystrophy patients and dystrophin-difficient mouse models [43-45]. Two products of up-regulated genes induced in Tg(HQK) muscle, CDNK1A (cyclin-dependent kinase inhibitor, p21) and GADD45B (growth arrest and DNA-damage inducible, beta), stand out as crucial to the initiation of cell cycle arrest mediated by activated p53. p53 tightly controls the expression of CDNK1A, which mediates the p53-dependent cell cycle arrest at the G1 phase by binding to and inhibiting the activity of cyclin-CDK2 or cyclin-CDK4 complexes in response to a variety of stress stimuli. Expression of CDNK1A was confirmed by qRT-PCR to be increased by more than 20-fold over that in control WT mice at 30 days post induction. The up-regulation of GADD45A, closely related in function to GADD45B, was also confirmed by qRT-PCR. These genes are often coordinately expressed and can function cooperatively to inhibit cell growth and induce apoptosis. Other up-regulated genes known to play a role in cell-cycle arrest are RB1, which binds to E2F transcription factors to prevent transcription of genes required for the G1 to S phase transition, and CGREF1, which is produced in response to stress and serves as a negative regulator of the cell cycle [46]. Taken together these gene expression changes indicate p53-dependent G1 cell cycle arrest was induced in Tg(HQK) muscle following induction of PrP^C ^expression.

Following cell cycle arrest, cells either recover or undergo p53-mediated apoptosis due to transcriptional activation of a number of pro-apoptotic genes. Key transducers of apoptosis include PMAIP1 (phorbol-12-myristate-13-acetate-induced protein 1 or Noxa) [47,48] and BBC3 (BCL2 binding component 3 or PUMA) [49,50]. Both were significantly up-regulated based on our microarray analysis. PMAIP1 induces the expression of other death effectors including BAK1 [51,52], which was also significantly induced in Dox-treated Tg(HQK) muscles. Deregulation of other apoptosis effector genes includes induction of the pro-death genes BOKI and the down-regulation of MCL1, a pro-survival BCL2 homologue. Numerous studies have identified the pro-apoptotic regulator BAX to be a major mediator of p53 induced apoptosis [53]. BAX was not identified as up-regulated by our microarray analysis because of the high cut-off value (> = 3-fold), but qRT-PCR revealed a modest up-regulation of the BAX gene (1.5–3.0 fold) over time following PrP over-expression. Similar to p53, TP73L (p63) can mediate apoptosis and was also found to be induced in atrophic muscles of Tg(HQK) mice. Less is known about the regulatory pathways triggered by p63 and its transcriptional targets have not been fully characterized [54-57]. Moreover, both the p53 apoptosis effector gene PERP and the p53-inducible ubiquitin ligase p53RFP (RNF144B) were significantly induced in the Tg(HQK) muscles as well. PERP is a potential marker of p53 driven apoptosis since it has been found to be induced in p53-driven apoptotic cells but not in p53-dependent G1 arrested cells and p53RFP has also been shown to be involved in switching a cell from p53-mediated growth arrest to apoptosis [58,59].

These data indicate that not only do muscle cells of Dox-treated Tg(HQK) mice undergo p53-dependent cell cycle arrest, but at least in some instances they go on to undergo apoptosis, which strongly suggests that p53-regulated pro-apoptotic pathways play an important role in PrP-mediated myopathy.

## Discussion

We have previously described the generation of the Tg(HQK) transgenic mice, in which Dox-induced over-expression of PrP^C ^specifically in the skeletal muscles causes a primary myopathy that is correlated with accumulation of an N-terminal truncated PrP C1 fragment [6]. The aim of this study was to determine the molecular basis for the PrP-mediated myopathy by microarray analysis. The ultimate goals are to fully understand the detailed molecular pathways of PrP-mediated myopathy, so that we can better understand the role of PrP in both normal and diseased muscles and provide some clues on the pathogenic mechanism of prion diseases. Utilizing two DNA microarrays, we identified more than 1000 genes that were temporally deregulated in a specific and highly consistent manner following induction of PrP^C ^over-expression in the muscles of Tg(HQK) mice and the subsequent development of myopathy. The transcriptional profiles in the muscles of Dox-treated Tg(HQK) mice strongly implicate toxicity-induced pro-apoptotic pathways in PrP-mediated myopathy, and they are quite different from the changes previously described in systemic, disuse, and denervation muscle atrophy.

Interestingly, the transcription factor MEF2C was found to be down-regulated at both the mRNA and protein levels in PrP^C^-mediated myopathy. MEF2C is expressed specifically in muscle and brain, where it is a target for signaling pathways involving calcium [60]. MEF2C regulates the expression of a majority of muscle-specific genes, and interacts with members of the MyoD family of proteins to activate muscle differentiation [29]. Calcium signaling was one of the pathways significantly induced in Dox-treated Tg(HQK) mouse muscles as evidenced by a very small p value of 8.75 × 10^-9^. The PrP^C ^protein has itself been shown to play a role in Ca^2+ ^homeostasis [61-63] and it is possible that over-expression of PrP^C ^results in perturbations in Ca^2+ ^signaling, which in turn modulates the activity and/or expression of MEF2C. As calcium regulation has also been shown to be altered during prion-induced neurodegeneration, this finding potentially links the molecular changes occurring in Tg(HQK) myopathy to the pathobiology of prion diseases.

The most striking finding is the strong and statistically highly significant induction of a p53-regulated pro-apoptotic network in Tg(HQK) mouse muscles following induction of PrP^C^. Central to this network are induction of p53 protein expression and strong induction of genes responsible for arresting the cell cycle, as well as a number of p53-regulated pro-apoptotic (up-regulated) and anti-apoptotic (down-regulated) genes. p53 is a critical tumor suppressor and transcription factor, and it has been linked to cell death in the central nervous system in a number of disorders including most notably neurodegenerative disorders such as Alzheimer's disease and prion diseases [64-66]. The expression of p53 protein has been found to rapidly increase in neurons in response to a range of insults including DNA damage, oxidative stress, metabolic compromise, and cellular calcium overload. Over-expression of PrP^C ^has been shown to enhance staurosporine-induced toxicity and activation of caspase-3 in the HEK293 kidney cell line [67] and increase sensitivity to apoptotic stimuli via p53-dependent pathways in TSM1 neuronal cell line [20]. Conversely neurons devoid of PrP^C ^expression were reported to display lower responsiveness to staurosporine, also via p53-dependent pathways [5].

One of the main pro-apoptotic effectors of p53 is BAX, which plays a major role in regulating neuronal death in the brain in response to a number of stimuli [68,69]. The role of BAX in prion-induced neurodegeneration is not well understood; both BAX-dependent and BAX-independent mechanisms appear to underlie the action of neurotoxic forms of prion proteins [70]. However, in the muscle of Dox-treated Tg(HQK) mice, only a marginal increase in BAX expression was observed whereas significant over-expression of other p53 regulated pro-apoptotic proteins, including BAK1, BBC3 and PMAIP1, and MCL1, were detected, suggesting that PrP^C^-mediated myopathy observed in this model may depend on Bax-independent pathways that involve BAK1, BBC3, PMAIP1, and MCL1.

**Figure 7:**
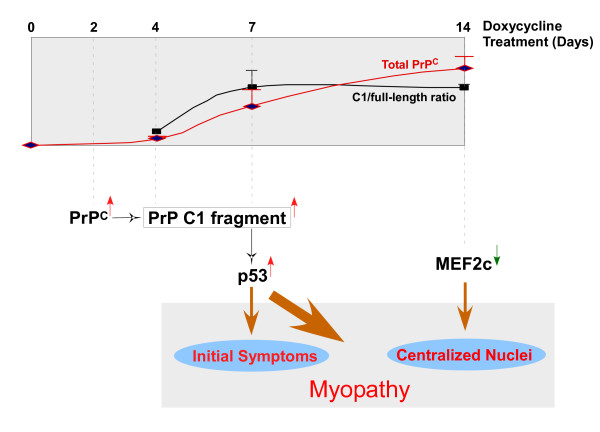
**Mechanism of PrP-mediated myopathy**. Accumulation of an N-terminal truncated PrP C1 fragment in muscle activates p53 resulting in the induction of p53-regulated pro-apoptotic networks and myopathic changes. PrP^C ^over-expression also results in down-regulation of MEF2C, which may be partially responsible for the progressive central nuclei localization observed in the muscles of Dox-treated Tg(HQK) mice.

We propose a working model to explain the mechanism of PrP-mediated myopathy (Figure 7). Dox-induced over-expression of PrP^C ^in the muscles leads to accumulation of the N-terminal truncated PrP C1 fragment, which in turn activates p53, thereby inducing p53-regulated pro-apoptotic networks and myopathic changes.

PrP accumulation has been observed in the skeletal muscles of patients with inclusion-body myositis, polymyositis, dermatomyositis, and neurogenic muscle atrophy, and we have previously reported that over-expression of wild type PrP in the skeletal muscles is sufficient to cause myopathy in the Tg(HQK) mice [[7] and references therein], which suggest that muscular accumulation of PrP may contribute to the pathogenesis of some human muscle diseases. Our finding that p53-related pathways play a major role in the myopathy in Tg(HQK) mice suggests that p53 and p53-related pathways may also be critical to the pathology of some human muscle disease patients and p53 and p53-related pathways may serve as potential targets for therapeutics development against these muscle diseases.

As we have previously reported [7], the preferential accumulation of the truncated PrP C1 fragment, which is generated through endoproteolysis of PrP^C ^during normal protein processing in the brain [8-12] and the muscle [7], was closely correlated with myopathic changes in Dox-treated Tg(HQK) mice. We hypothesize that it is this C1 fragment that is the toxic species in the Tg(HQK) model, which is supported by recent reports showing that over-expression of the C1 fragment increases cell death and caspase-3 activity through a p53-dependent mechanism [20,71]. Truncation of PrP^C ^occurs between residues 110 and 111 within a region shown to play a pivotal role in its conformational transition to PrP^Sc^. So a better understanding of modulation of this cleavage event and the mechanism for the truncated PrP fragments as mediators of a toxic cellular response may be very important in dissecting prion disease pathogenesis.

## Conclusion

In summary, we used microarrays to determine the molecular mechanism that underlies the myopathy observed in PrP over-expressing mice. The transcriptional changes induced in the Dox-treated Tg(HQK) mice are quite different from the changes previously described in systemic diseases and disuse and denervation atrophy. Significantly we found that the p53 protein and p53-regulated pro-apoptotic pathways are highly activated in the muscles of doxycycline-treated Tg(HQK) mice, correlating well with the observed myopathic changes. To our best knowledge, this is the first in vivo evidence that directly links p53 to a wild type PrP-mediated disease. We hypothesize that it is the preferentially accumulated truncated C1 fragment in the muscles of doxycycline-treated Tg(HQK) mice that activates the p53 pathway, resulting in the primary myopathy. This is consistent with recent reports showing that over-expression of the C1 fragment increase cell death and caspase-3 activity through a p53-dependent mechanism in cell culture models.

Dissecting how PrP regulates the p53 pathways may help understand PrP-mediated pathogenesis in both muscle diseases and prion diseases. Neuronal loss, a salient feature of prion diseases, has been reported to be due to neuronal apoptosis in prion-affected humans and animals [72-75]. p53 has been shown to be a critical player in PrP or PrP fragments-mediated cytotoxicity in neurons [5,20-22]. Therefore, our finding that p53 plays a major role in PrP-mediated myopathy and our future follow-up studies on the detailed molecular mechanisms of how PrP over-expression leads to p53 activation in the muscles, may also provide some clues on the molecular mechanism of prion pathogenesis in the brain.

## Authors' contributions

SAB and QK conceived the work and jointly participated in its design and coordination. JL performed the animal experiments and immunoblots and, along with SAB and QK, participated in drafting the manuscript. QK provided tissue samples for transcriptional profiling, and SH and MW provided the early-stage characterization and care for the animals. SM, GS and LL performed the RNA extractions, microarrays and RT-PCR validation. SAB and DP coordinated and performed all the bioinformatic and statistical analyses of array data. All authors read and approved the final manuscript.

## Supplementary Material

Additional File 1 **Genes up-regulated in Tg(HQK) muscle following induction of PrP over-expression**. The data provided represents a list of genes determined to be up-regulated following induction of PrP over-expression in Tg(HQK) muscle. Genes include those found to be temporally de-regulated on the BMAP platform and those found using the Agilent microarray platform at 14 days post induction. Click here for file

Additional File 2 **Genes down-regulated in Tg(HQK) muscle following induction of PrP over-expression**. The data provided represents a list of genes determined to be down-regulated following induction of PrP over-expression in Tg(HQK) muscle. Genes include those found to be temporally de-regulated on the BMAP platform and those found using the Agilent microarray platform at 14 days post induction. Click here for file
