# Supplementary material for: Activation of p53-regulated pro-apoptotic signaling pathways in PrP-mediated myopathy
Source: BMC Genomics. 2009 Apr 28;10:201. doi: 10.1186/1471-2164-10-201 (PMC2683871; doi:10.1186/1471-2164-10-201)
Supplement: Additional File 1 — Genes up-regulated in Tg(HQK) muscle following induction of PrP over-expression. The data provided represents a list of genes determined to be up-regulated following induction of PrP over-expression in Tg(HQK) muscle. Genes include those found to be temporally de-regulated on the BMAP platform and those found using the Agilent microarray platform at 14 days post induction. [file 1471-2164-10-201-S1.doc]

|  |  |  |  | |  |
| --- | --- | --- | --- | --- | --- |
| **Name** | **Description** | **GenBank** | **Fold Change** | | |
|  |  |  |  | |  |
|  |  |  |  |  | |
| SEPT5 | septin 5 | NM_213614 | 3.702 |  | |
| SEPT9 | septin 9 | NM_017380 | 3.522 |  | |
| SEPT11 | septin 11 | AI836723 | * |  | |
| 2310043N10RIK | RIKEN cDNA 2310043N10 gene | AK028745 | 3.292 |  | |
| A2BP1 | ataxin 2-binding protein 1 | AI835398 | * |  | |
| ABHD2 | abhydrolase domain containing 2 | NM_018811 | 11.883 |  | |
| ABI2 | abl interactor 2 | NM_198127 | 3.075 |  | |
| ABL1 | v-abl Abelson murine leukemia viral oncogene homolog 1 | AI325092 | * |  | |
| ACBD3 | acyl-Coenzyme A binding domain containing 3 | AI450805 | * |  | |
| ACCN3 | amiloride-sensitive cation channel 3 | NM_183000 | 3.276 |  | |
| ACHE | acetylcholinesterase (Yt blood group) | NM_009599 | 3.274 |  | |
| ACOT9 | acyl-CoA thioesterase 9 | AI427919 | * |  | |
| ACTR3B | ARP3 actin-related protein 3 homolog B (yeast) | BC053106 | 8.554 |  | |
| ADAM8 | ADAM metallopeptidase domain 8 | NM_007403 | 11.279 |  | |
| ADCY7 | adenylate cyclase 7 | AK012436 | 5.258 |  | |
| ADIPOR2 | adiponectin receptor 2 | AI854179 | * |  | |
| ADPGK | ADP-dependent glucokinase | NM_028121 | 4.142 |  | |
| ADPRHL2 | ADP-ribosylhydrolase like 2 | AI836109 | * |  | |
| ADSSL1 | adenylosuccinate synthase like 1 | AI848125 | * |  | |
| AFAP1 | actin filament associated protein 1 | AI848729 | * |  | |
| AFMID | arylformamidase | AI837760 | * |  | |
| AGPAT2 | 1-acylglycerol-3-phosphate O-acyltransferase 2 (lysophosphatidic acid acyltransferase, beta) | AI835177 | * |  | |
| AGPAT3 | 1-acylglycerol-3-phosphate O-acyltransferase 3 | AI449684 | * |  | |
| AGXT2 | alanine-glyoxylate aminotransferase 2 | AI464452 | * |  | |
| AIF1 | allograft inflammatory factor 1 | NM_019467 | 3.632 |  | |
| AK2 | adenylate kinase 2 | AI854898 | * |  | |
| AK3 | adenylate kinase 3 | AI854743 | * |  | |
| AKR1C2 | aldo-keto reductase family 1, member C2 (dihydrodiol dehydrogenase 2; bile acid binding protein; 3-alpha hydroxysteroid dehydrogenase, type III) | AI323755 | * |  | |
| AKT1S1 | AKT1 substrate 1 (proline-rich) | AI838420 | * |  | |
| ALS2CR2 | amyotrophic lateral sclerosis 2 (juvenile) chromosome region, candidate 2 | AI853959 | * |  | |
| AMFR | autocrine motility factor receptor | AI847084 | * |  | |
| ANAPC1 | anaphase promoting complex subunit 1 | AK053383 | 3.24 |  | |
| ANKRD1 | ankyrin repeat domain 1 (cardiac muscle) | NM_013468 | 4.331 |  | |
| ANKRD10 | ankyrin repeat domain 10 | NM_133971 | 4.108 |  | |
| ANLN | anillin, actin binding protein | AI429324 | * |  | |
| ANXA2 | annexin A2 | AK087259 | 6.952 |  | |
| ANXA4 | annexin A4 | NM_013471 | 3.719 |  | |
| ANXA5 | annexin A5 | AI893564 | * |  | |
| ANXA6 | annexin A6 | AK077843 | 5.107 |  | |
| ANXA7 | annexin A7 | AK032013 | 6.168 |  | |
| AP1G1 | adaptor-related protein complex 1, gamma 1 subunit | AK037086 | 3.589 |  | |
| AP1G2 | adaptor-related protein complex 1, gamma 2 subunit | NM_007455 | 3.292 |  | |
| AP1M1 | adaptor-related protein complex 1, mu 1 subunit | AI854661 | * |  | |
| AP2B1 | adaptor-related protein complex 2, beta 1 subunit | AI845018 | * |  | |
| APLP2 | amyloid beta (A4) precursor-like protein 2 | AI849502 | * |  | |
| APOBEC1 | apolipoprotein B mRNA editing enzyme, catalytic polypeptide 1 | NM_031159 | 3.164 |  | |
| APOC1 | apolipoprotein C-I | AI893652 | * |  | |
| APP | amyloid beta (A4) precursor protein (peptidase nexin-II, Alzheimer disease) | AK030583 | 4.203 |  | |
| AQP7 | aquaporin 7 | AI429089 | * |  | |
| ARF6 | ADP-ribosylation factor 6 | AI854231 | * |  | |
| ARFGAP3 | ADP-ribosylation factor GTPase activating protein 3 | NM_025445 | 4.216 |  | |
| ARHGAP12 | Rho GTPase activating protein 12 | AI465207 | * |  | |
| ARHGAP22 | Rho GTPase activating protein 22 | NM_153800 | 3.792 |  | |
| ARHGAP5 | Rho GTPase activating protein 5 | AK080589 | 3.407 |  | |
| ARHGDIB | Rho GDP dissociation inhibitor (GDI) beta | AI323807 | * |  | |
| ARHGDIG | Rho GDP dissociation inhibitor (GDI) gamma | NM_008113 | 3.675 |  | |
| ARHGEF12 | Rho guanine nucleotide exchange factor (GEF) 12 | BC052785 | 3.265 |  | |
| ARID1B | AT rich interactive domain 1B (SWI1-like) | AI853672 | * |  | |
| ARID3A | AT rich interactive domain 3A (BRIGHT-like) | AI528789 | * |  | |
| ARL4C | ADP-ribosylation factor-like 4C | NM_177305 | 3.365 |  | |
| ARL6IP4 | ADP-ribosylation-like factor 6 interacting protein 4 | AI464443 | * |  | |
| ARPC4 | actin related protein 2/3 complex, subunit 4, 20kDa | AI327076 | * |  | |
| ASB2 | ankyrin repeat and SOCS box-containing 2 | AI842575 | * |  | |
| ASGR1 | asialoglycoprotein receptor 1 | AI845829 | * |  | |
| ATG4B | ATG4 autophagy related 4 homolog B (S. cerevisiae) | AI464444 | * |  | |
| ATIC | 5-aminoimidazole-4-carboxamide ribonucleotide formyltransferase/IMP cyclohydrolase | AI835570 | * |  | |
| ATOX1 | ATX1 antioxidant protein 1 homolog (yeast) | AI464567 | * |  | |
| ATP13A5 | ATPase type 13A5 | NM_175650 | 7.679 |  | |
| ATP2A2 | ATPase, Ca++ transporting, cardiac muscle, slow twitch 2 | AI854716 | * |  | |
| ATP6V0A1 | ATPase, H+ transporting, lysosomal V0 subunit a1 | AK045877 | 3.283 |  | |
| ATP6V0D1 | ATPase, H+ transporting, lysosomal 38kDa, V0 subunit d1 | AI849250 | * |  | |
| ATXN2 | ataxin 2 | AK036209 | 4.376 |  | |
| AYTL2 | acyltransferase like 2 | AI847713 | * |  | |
| B4GALNT1 | beta-1,4-N-acetyl-galactosaminyl transferase 1 | NM_008080 | 4.663 |  | |
| BAIAP2 | BAI1-associated protein 2 | NM_130862 | 4.238 |  | |
| BAK1 | BCL2-antagonist/killer 1 | NM_007523 | 3.049 |  | |
| BBC3 | BCL2 binding component 3 | NM_133234 | 3.001 |  | |
| BAZ1A | bromodomain adjacent to zinc finger domain, 1A | XM_885873 | 4.364 |  | |
| BCL2A1 | BCL2-related protein A1 | NM_007534 | 6.716 |  | |
| BCL2L13 | BCL2-like 13 (apoptosis facilitator) | AI853789 | * |  | |
| BDP1 | B double prime 1, subunit of RNA polymerase III transcription initiation factor IIIB | AI450927 | * |  | |
| BIN1 | bridging integrator 1 | NM_009668 | 3.071 |  | |
| BIRC5 | baculoviral IAP repeat-containing 5 (survivin) | NM_009689 | 3.408 |  | |
| BLZF1 | basic leucine zipper nuclear factor 1 (JEM-1) | AI428215 | * |  | |
| BMPR1B | bone morphogenetic protein receptor, type IB | AI464533 | * |  | |
| BMPR2 | bone morphogenetic protein receptor, type II (serine/threonine kinase) | AK083553 | 3.433 |  | |
| BNIP1 | BCL2/adenovirus E1B 19kDa interacting protein 1 | AK170908 | 3.271 |  | |
| BOK | BCL2-related ovarian killer | NM_016778 | 3.218 |  | |
| BPTF | bromodomain PHD finger transcription factor | AI851639 | * |  | |
| BRAF | v-raf murine sarcoma viral oncogene homolog B1 | AI426984 | * |  | |
| C13ORF24 | chromosome 13 open reading frame 24 | AK045133 | 3.185 |  | |
| C14ORF130 | chromosome 14 open reading frame 130 | AI851583 | * |  | |
| C1ORF57 | chromosome 1 open reading frame 57 | AI449709 | * |  | |
| C1QBP | complement component 1, q subcomponent binding protein | AI464355 | * |  | |
| C9ORF52 | chromosome 9 open reading frame 52 | AI450603 | * |  | |
| C9ORF72 | chromosome 9 open reading frame 72 | AI835604 | * |  | |
| CABIN1 | calcineurin binding protein 1 | AI427692 | * |  | |
| CACNA2D4 | calcium channel, voltage-dependent, alpha 2/delta subunit 4 | AK030723 | 4.497 |  | |
| CAMK1D | calcium/calmodulin-dependent protein kinase ID | AK141415 | 3.666 |  | |
| CAMK2N2 | calcium/calmodulin-dependent protein kinase II inhibitor 2 | AK013788 | 3.172 |  | |
| CAPRIN1 | cell cycle associated protein 1 | AK051363 | 3.329 |  | |
| CARD10 | caspase recruitment domain family, member 10 | AI449026 | * |  | |
| CARD11 | caspase recruitment domain family, member 11 | NM_175362 | 3.961 |  | |
| CARHSP1 | calcium regulated heat stable protein 1, 24kDa | AI449676 | * |  | |
| CASD1 | CAS1 domain containing 1 | AK087204 | 3.051 |  | |
| CASP14 | caspase 14, apoptosis-related cysteine peptidase | AI448765 | * |  | |
| CBX5 | chromobox homolog 5 (HP1 alpha homolog, Drosophila) | AI893653 | * |  | |
| CBX6 | chromobox homolog 6 | AI847596 | * |  | |
| CCL7 | chemokine (C-C motif) ligand 7 | NM_013654 | 3.259 |  | |
| CCL8 | chemokine (C-C motif) ligand 8 | NM_021443 | 4.678 |  | |
| CCRK | cell cycle related kinase | AI326314 | * |  | |
| CD28 | CD28 molecule | AI327367 | * |  | |
| CD300D | Cd300D antigen | NM_134158 | 4.528 |  | |
| CD44 | CD44 molecule (Indian blood group) | NM_009851 | 3.216 |  | |
| CD52 | CD52 molecule | NM_013706 | 4.952 |  | |
| CD81 | CD81 molecule | AI840865 | * |  | |
| CD82 | CD82 molecule | NM_007656 | 3.103 |  | |
| CDC14A | CDC14 cell division cycle 14 homolog A (S. cerevisiae) | XM_901214 | 6.529 |  | |
| CDC27 | cell division cycle 27 homolog (S. cerevisiae) | AI452358 | * |  | |
| CDC34 | cell division cycle 34 homolog (S. cerevisiae) | AI465204 | * |  | |
| CDK5R1 | cyclin-dependent kinase 5, regulatory subunit 1 (p35) | NM_009871 | 8.378 |  | |
| CDK6 | cyclin-dependent kinase 6 | AK030810 | 5.275 |  | |
| CDKN1A | cyclin-dependent kinase inhibitor 1A (p21, Cip1) | NM_007669 | 3.124 |  | |
| CDS1 | CDP-diacylglycerol synthase (phosphatidate cytidylyltransferase) 1 | AI844295 | * |  | |
| CDS2 | CDP-diacylglycerol synthase (phosphatidate cytidylyltransferase) 2 | BC059776 | 6.212 |  | |
| CENPA | centromere protein A | AI449898 | * |  | |
| CENPO | centromere protein O | AI448222 | * |  | |
| CES2 (includes EG:234671) | carboxylesterase 2 | NM_145603 | 4.611 |  | |
| CGREF1 | cell growth regulator with EF-hand domain 1 | BC023116 | 6.022 |  | |
| CHD2 | chromodomain helicase DNA binding protein 2 | AI449389 | * |  | |
| CHES1 | checkpoint suppressor 1 | AI849096 | * |  | |
| CHKB | choline kinase beta | AI326292 | * |  | |
| CHM | choroideremia (Rab escort protein 1) | AI447929 | * |  | |
| CHP | calcium binding protein P22 | AI845888 | * |  | |
| CHRNA1 | cholinergic receptor, nicotinic, alpha 1 (muscle) | NM_007389 | 5.735 |  | |
| CHRNB4 | cholinergic receptor, nicotinic, beta 4 | AI845506 | * |  | |
| CHRNG | cholinergic receptor, nicotinic, gamma | NM_009604 | 17.62 |  | |
| CHST11 | carbohydrate (chondroitin 4) sulfotransferase 11 | NM_021439 | 4.13 |  | |
| CIC | capicua homolog (Drosophila) | AI836798 | * |  | |
| CIITA | class II, major histocompatibility complex, transactivator | NM_007575 | 3.559 |  | |
| CISH | cytokine inducible SH2-containing protein | NM_009895 | 10.814 |  | |
| CITED2 | Cbp/p300-interacting transactivator, with Glu/Asp-rich carboxy-terminal domain, 2 | U86445 | 4.376 |  | |
| CKAP4 | cytoskeleton-associated protein 4 | NM_175451 | 4.842 |  | |
| CLASP1 | cytoplasmic linker associated protein 1 | AK080782 | 3.099 |  | |
| CLDN8 | claudin 8 | AI450616 | * |  | |
| CLEC7A | C-type lectin domain family 7, member A | NM_020008 | 3.662 |  | |
| CLINT1 | clathrin interactor 1 | AI427041 | * |  | |
| CMPK | cytidylate kinase | AI836710 | * |  | |
| CNDP1 | carnosine dipeptidase 1 (metallopeptidase M20 family) | AI427125 | * |  | |
| CNN3 | calponin 3, acidic | AI843550 | * |  | |
| COL19A1 | collagen, type XIX, alpha 1 | NM_007733 | 5.043 |  | |
| COL1A2 | collagen, type I, alpha 2 | AI841886 | * |  | |
| COL4A2 | collagen, type IV, alpha 2 | AI843282 | * |  | |
| COL5A3 | collagen, type V, alpha 3 | NM_016919 | 5.685 |  | |
| COL7A1 | collagen, type VII, alpha 1 (epidermolysis bullosa, dystrophic, dominant and recessive) | NM_007738 | 7.27 |  | |
| COPS7A | COP9 constitutive photomorphogenic homolog subunit 7A (Arabidopsis) | AI427951 | * |  | |
| COTL1 | coactosin-like 1 (Dictyostelium) | NM_028071 | 3.837 |  | |
| CPEB1 | cytoplasmic polyadenylation element binding protein 1 | NM_007755 | 3.326 |  | |
| CRKL | v-crk sarcoma virus CT10 oncogene homolog (avian)-like | AI325100 | * |  | |
| CSF2RA | colony stimulating factor 2 receptor, alpha, low-affinity (granulocyte-macrophage) | NM_009970 | 5.003 |  | |
| CSK | c-src tyrosine kinase | AI324651 | * |  | |
| CSPG4 | chondroitin sulfate proteoglycan 4 | NM_139001 | 3.443 |  | |
| CST6 | cystatin E/M | AI853055 | * |  | |
| CTDSPL | CTD (carboxy-terminal domain, RNA polymerase II, polypeptide A) small phosphatase-like | AI464471 | * |  | |
| CTF1 | cardiotrophin 1 | AI893883 | * |  | |
| CTSD | cathepsin D | NM_009983 | 3.003 |  | |
| CTSS | cathepsin S | NM_021281 | 4.606 |  | |
| CTSZ | cathepsin Z | NM_022325 | 3.126 |  | |
| CTTN | cortactin | AI853713 | * |  | |
| CX3CL1 | chemokine (C-X3-C motif) ligand 1 | NM_009142 | 3.377 |  | |
| CXADR | coxsackie virus and adenovirus receptor | AI427058 | * |  | |
| CXXC4 | CXXC finger 4 | AI845873 | * |  | |
| CYB5R3 | cytochrome b5 reductase 3 | NM_029787 | 3.371 |  | |
| DAB1 | disabled homolog 1 (Drosophila) | AI427087 | * |  | |
| DAG1 | dystroglycan 1 (dystrophin-associated glycoprotein 1) | AI325151 | * |  | |
| DAP3 | death associated protein 3 | AI464536 | * |  | |
| DCLK3 | doublecortin-like kinase 3 | AI853072 | * |  | |
| DCX | doublecortex; lissencephaly, X-linked (doublecortin) | AI843371 | * |  | |
| DDAH1 | dimethylarginine dimethylaminohydrolase 1 | AI661738 | * |  | |
| DDEF1 | development and differentiation enhancing factor 1 | XM_976949 | 3.326 |  | |
| DDOST (includes EG:1650) | dolichyl-diphosphooligosaccharide-protein glycosyltransferase | AI852284 | * |  | |
| DDX3Y (includes EG:26900) | DEAD (Asp-Glu-Ala-Asp) box polypeptide 3, Y-linked | NM_012008 | 276.925 |  | |
| DEDD | death effector domain containing | AI853070 | * |  | |
| DGKZ | diacylglycerol kinase, zeta 104kDa | NM_138306 | 3.272 |  | |
| DHX16 | DEAH (Asp-Glu-Ala-His) box polypeptide 16 | AI449975 | * |  | |
| DHX36 | DEAH (Asp-Glu-Ala-His) box polypeptide 36 | AI465406 | * |  | |
| DIABLO | diablo homolog (Drosophila) | BC024780 | 3.58 |  | |
| DIAPH1 | diaphanous homolog 1 (Drosophila) | BC070412 | 3.902 |  | |
| DLGAP4 | discs, large (Drosophila) homolog-associated protein 4 | NM_146128 | 3.53 |  | |
| DLST | dihydrolipoamide S-succinyltransferase (E2 component of 2-oxo-glutarate complex) | AK171100 | 3.33 |  | |
| DMP1 | dentin matrix acidic phosphoprotein | NM_016779 | 8.683 |  | |
| DNAJC10 | DnaJ (Hsp40) homolog, subfamily C, member 10 | AI852588 | * |  | |
| DNAJC3 | DnaJ (Hsp40) homolog, subfamily C, member 3 | NM_008929 | 4.502 |  | |
| DOCK1 | dedicator of cytokinesis 1 | AI854900 | * |  | |
| DPEP2 | dipeptidase 2 | NM_176913 | 4.871 |  | |
| DPP3 | dipeptidyl-peptidase 3 | AK039975 | 4.533 |  | |
| DTL | denticleless homolog (Drosophila) | NM_029766 | 4.05 |  | |
| DTNB | dystrobrevin, beta | NM_007886 | 3.713 |  | |
| DUSP10 | dual specificity phosphatase 10 | AK080157 | 5.468 |  | |
| DUSP2 | dual specificity phosphatase 2 | NM_010090 | 3.039 |  | |
| DUSP4 | dual specificity phosphatase 4 | NM_176933 | 6.609 |  | |
| DYNC1H1 | dynein, cytoplasmic 1, heavy chain 1 | Z83808 | 3.421 |  | |
| DYNC1LI2 | dynein, cytoplasmic 1, light intermediate chain 2 | AI851670 | * |  | |
| DYNC2LI1 | dynein, cytoplasmic 2, light intermediate chain 1 | AI464530 | * |  | |
| DYSF | dysferlin, limb girdle muscular dystrophy 2B (autosomal recessive) | NM_021469 | 3.259 |  | |
| E2F1 | E2F transcription factor 1 | NM_007891 | 3.586 |  | |
| E2F2 | E2F transcription factor 2 | NM_177733 | 4.5 |  | |
| E2F3 | E2F transcription factor 3 | NM_010093 | 3.176 |  | |
| E2F8 | E2F transcription factor 8 | NM_001013368 | 3.488 |  | |
| EDA2R | ectodysplasin A2 receptor | AK029268 | 7.405 |  | |
| EDEM1 | ER degradation enhancer, mannosidase alpha-like 1 | NM_138677 | 3.156 |  | |
| EDF1 | endothelial differentiation-related factor 1 | AI836001 | * |  | |
| EEF1A1 | eukaryotic translation elongation factor 1 alpha 1 | AI835939 | * |  | |
| EGFBP2 | epidermal growth factor binding protein type B | NM_010115 | 3.646 |  | |
| EGR1 | early growth response 1 | NM_007913 | 14.613 |  | |
| EHD4 | EH-domain containing 4 | AI846352 | * |  | |
| EHMT2 | euchromatic histone-lysine N-methyltransferase 2 | AI835615 | * |  | |
| EIF2S1 | eukaryotic translation initiation factor 2, subunit 1 alpha, 35kDa | AI427024 | * |  | |
| EIF2S3 | eukaryotic translation initiation factor 2, subunit 3 gamma, 52kDa | NM_012010 | 4.258 |  | |
| EIF2S3Y | eukaryotic translation initiation factor 2, subunit 3, structural gene Y-linked | NM_012011 | 62.424 |  | |
| EIF3S10 | eukaryotic translation initiation factor 3, subunit 10 theta, 150/170kDa | AI836309 | * |  | |
| EIF3S12 | eukaryotic translation initiation factor 3, subunit 12 | AI840840 | * |  | |
| EIF3S7 | eukaryotic translation initiation factor 3, subunit 7 zeta, 66/67kDa | AI326172 | * |  | |
| ELN | elastin (supravalvular aortic stenosis, Williams-Beuren syndrome) | AI385707 | * |  | |
| ELOVL3 | elongation of very long chain fatty acids (FEN1/Elo2, SUR4/Elo3, yeast)-like 3 | NM_007703 | 7.58 |  | |
| ELOVL6 | ELOVL family member 6, elongation of long chain fatty acids (FEN1/Elo2, SUR4/Elo3-like, yeast) | AI465340 | * |  | |
| ELP3 | elongation protein 3 homolog (S. cerevisiae) | AI851259 | * |  | |
| EMP3 | epithelial membrane protein 3 | AI851537 | * |  | |
| ENAH | enabled homolog (Drosophila) | NM_010135 | 3.361 |  | |
| ENO2 | enolase 2 (gamma, neuronal) | NM_013509 | 5.128 |  | |
| EPB41L2 | erythrocyte membrane protein band 4.1-like 2 | AI837696 | * |  | |
| EPHB1 | EPH receptor B1 | NM_173447 | 3.677 |  | |
| ERBB2IP | erbb2 interacting protein | AK045806 | 4.378 |  | |
| ESRRG | estrogen-related receptor gamma | AI426536 | * |  | |
| EXOC3 | exocyst complex component 3 | AI464559 | * |  | |
| EXOC6B | exocyst complex component 6B | AI448736 | * |  | |
| EXOSC7 | exosome component 7 | AI851760 | * |  | |
| EXTL1 | exostoses (multiple)-like 1 | AI850861 | * |  | |
| F10 | coagulation factor X | NM_007972 | 10.84 |  | |
| F2 | coagulation factor II (thrombin) | NM_010168 | 6.481 |  | |
| F2R | coagulation factor II (thrombin) receptor | AI464520 | * |  | |
| F7 | coagulation factor VII (serum prothrombin conversion accelerator) | NM_010172 | 5.124 |  | |
| FADS3 | fatty acid desaturase 3 | AI464531 | * |  | |
| FAF1 | Fas (TNFRSF6) associated factor 1 | AI843154 | * |  | |
| FAM110B | family with sequence similarity 110, member B | AI449876 | * |  | |
| FAM46A | family with sequence similarity 46, member A | AI447893 | * |  | |
| FARP2 | FERM, RhoGEF and pleckstrin domain protein 2 | AI465173 | * |  | |
| FBXO3 | F-box protein 3 | AI428262 | * |  | |
| FBXW11 | F-box and WD repeat domain containing 11 | AK010033 | 3.478 |  | |
| FCER1G | Fc fragment of IgE, high affinity I, receptor for; gamma polypeptide | AI573376 | * |  | |
| FCGR1A | Fc fragment of IgG, high affinity Ia, receptor (CD64) | NM_010186 | 3.881 |  | |
| FDPS | farnesyl diphosphate synthase (farnesyl pyrophosphate synthetase, dimethylallyltranstransferase, geranyltranstransferase) | AK077979 | 3.024 |  | |
| FEM1A | fem-1 homolog a (C. elegans) | AI836048 | * |  | |
| FEM1C | fem-1 homolog c (C. elegans) | AK031720 | 4.242 |  | |
| FEZ1 | fasciculation and elongation protein zeta 1 (zygin I) | NM_183171 | 4.867 |  | |
| FEZ2 | fasciculation and elongation protein zeta 2 (zygin II) | AI853949 | * |  | |
| FFAR2 | free fatty acid receptor 2 | NM_146187 | 3.532 |  | |
| FGD6 | FYVE, RhoGEF and PH domain containing 6 | AI427095 | * |  | |
| FGF21 | fibroblast growth factor 21 | NM_020013 | 5.711 |  | |
| FHIT | fragile histidine triad gene | AI465200 | * |  | |
| FILIP1L | filamin A interacting protein 1-like | AI464359 | * |  | |
| FKBP11 | FK506 binding protein 11, 19 kDa | NM_024169 | 5.175 |  | |
| FLNC | filamin C, gamma (actin binding protein 280) | BC060276 | 3.057 |  | |
| FN1 | fibronectin 1 | BC010335 | 6.08 |  | |
| FOS | v-fos FBJ murine osteosarcoma viral oncogene homolog | NM_010234 | 7.535 |  | |
| FOSL1 | FOS-like antigen 1 | NM_010235 | 3.774 |  | |
| FOXK2 | forkhead box K2 | AK078027 | 4.187 |  | |
| FOXP1 | forkhead box P1 | AI851964 | * |  | |
| FRRS1 | ferric-chelate reductase 1 | NM_009146 | 3.004 |  | |
| FSCN1 | fascin homolog 1, actin-bundling protein (Strongylocentrotus purpuratus) | AI847447 | * |  | |
| FXYD7 | FXYD domain containing ion transport regulator 7 | AI849202 | * |  | |
| G3BP1 | GTPase activating protein (SH3 domain) binding protein 1 | AI850849 | * |  | |
| GABARAPL1 | GABA(A) receptor-associated protein like 1 | AI465443 | * |  | |
| GABRA6 | gamma-aminobutyric acid (GABA) A receptor, alpha 6 | AI839865 | * |  | |
| GADD45B | growth arrest and DNA-damage-inducible, beta | AI848850 | * |  | |
| GADD45G | growth arrest and DNA-damage-inducible, gamma | AI839475 | * |  | |
| GAK | cyclin G associated kinase | AI835148 | * |  | |
| GALNT6 | UDP-N-acetyl-alpha-D-galactosamine:polypeptide N-acetylgalactosaminyltransferase 6 (GalNAc-T6) | NM_172451 | 3.54 |  | |
| GAPDH (includes EG:14433) | glyceraldehyde-3-phosphate dehydrogenase | AI835452 | * |  | |
| GATM | glycine amidinotransferase (L-arginine:glycine amidinotransferase) | AI844626 | * |  | |
| GBAS | glioblastoma amplified sequence | AI854717 | * |  | |
| GBL | G protein beta subunit-like | AI448158 | * |  | |
| GCDH | glutaryl-Coenzyme A dehydrogenase | AI844454 | * |  | |
| GCSH | glycine cleavage system protein H (aminomethyl carrier) | AI851345 | * |  | |
| GDF11 | growth differentiation factor 11 | XM_125935 | 4.543 |  | |
| GDF15 | growth differentiation factor 15 | NM_011819 | 4.284 |  | |
| GDNF | glial cell derived neurotrophic factor | NM_010275 | 4.928 |  | |
| GFER | growth factor, augmenter of liver regeneration (ERV1 homolog, S. cerevisiae) | AI851638 | * |  | |
| GHR | growth hormone receptor | AK053579 | 3.926 |  | |
| GJA3 | gap junction protein, alpha 3, 46kDa | AK136383 | 6.297 |  | |
| GKAP1 | G kinase anchoring protein 1 | AI847897 | * |  | |
| GLIS1 | GLIS family zinc finger 1 | NM_147221 | 3.852 |  | |
| GLS | glutaminase | AK047664 | 4.069 |  | |
| GLTSCR2 | glioma tumor suppressor candidate region gene 2 | AI840938 | * |  | |
| GNA13 | guanine nucleotide binding protein (G protein), alpha 13 | AI426467 | * |  | |
| GNAS | GNAS complex locus | AK017423 | 3.209 |  | |
| GNG3 | guanine nucleotide binding protein (G protein), gamma 3 | AI836851 | * |  | |
| GOLGA3 | golgi autoantigen, golgin subfamily a, 3 | AI449376 | * |  | |
| GP1BB | glycoprotein Ib (platelet), beta polypeptide | AI840315 | * |  | |
| GPATCH8 | G patch domain containing 8 | AI854807 | * |  | |
| GPM6B | glycoprotein M6B | AI843757 | * |  | |
| GPNMB | glycoprotein (transmembrane) nmb | NM_053110 | 8.701 |  | |
| GPR77 | G protein-coupled receptor 77 | NM_176912 | 4.124 |  | |
| GPT | glutamic-pyruvate transaminase (alanine aminotransferase) | AI427571 | * |  | |
| GPX1 | glutathione peroxidase 1 | AI847258 | * |  | |
| GREB1 | GREB1 protein | AI447633 | * |  | |
| GSK3B | glycogen synthase kinase 3 beta | AI848867 | * |  | |
| GSR | glutathione reductase | AI854660 | * |  | |
| GSTA5 | glutathione S-transferase A5 | NM_008181 | 15.402 |  | |
| GSTT2 | glutathione S-transferase theta 2 | AI448400 | * |  | |
| GTF3C5 | general transcription factor IIIC, polypeptide 5, 63kDa | AI449033 | * |  | |
| GTL2 | GTL2, imprinted maternally expressed untranslated mRNA | NM_144513 | 3.966 |  | |
| HADH | hydroxyacyl-Coenzyme A dehydrogenase | AI846363 | * |  | |
| HBD | hemoglobin, delta | AI323916 | * |  | |
| HBEGF | heparin-binding EGF-like growth factor | NM_010415 | 4.611 |  | |
| HBG1 | hemoglobin, gamma A | AI385765 | * |  | |
| HBP1 | HMG-box transcription factor 1 | AI465172 | * |  | |
| HCCA2 | HCCA2 protein | AI841931 | * |  | |
| HDAC5 | histone deacetylase 5 | AI426555 | * |  | |
| HERPUD1 | homocysteine-inducible, endoplasmic reticulum stress-inducible, ubiquitin-like domain member 1 | AI835088 | * |  | |
| HEXA | hexosaminidase A (alpha polypeptide) | AI842949 | * |  | |
| HEXB | hexosaminidase B (beta polypeptide) | AI854206 | * |  | |
| HIPK2 | homeodomain interacting protein kinase 2 | AK016742 | 4.238 |  | |
| HIPK3 | homeodomain interacting protein kinase 3 | AI449703 | * |  | |
| HISPPD2A | histidine acid phosphatase domain containing 2A | AK160847 | 3.224 |  | |
| HIVEP3 | human immunodeficiency virus type I enhancer binding protein 3 | AK038070 | 3.886 |  | |
| HLA-DMB | major histocompatibility complex, class II, DM beta | NM_010387 | 3.012 |  | |
| HLA-DOB | major histocompatibility complex, class II, DO beta | AI326012 | * |  | |
| HMBS | hydroxymethylbilane synthase | AI853649 | * |  | |
| HMGCS2 | 3-hydroxy-3-methylglutaryl-Coenzyme A synthase 2 (mitochondrial) | AI324952 | * |  | |
| HMGN2 | high-mobility group nucleosomal binding domain 2 | AI836705 | * |  | |
| HNRPLL | heterogeneous nuclear ribonucleoprotein L-like | AI852082 | * |  | |
| HNRPM | heterogeneous nuclear ribonucleoprotein M | AI851185 | * |  | |
| HOOK1 | hook homolog 1 (Drosophila) | AI464383 | * |  | |
| HOOK3 | hook homolog 3 (Drosophila) | AI851190 | * |  | |
| HPR | haptoglobin-related protein | NM_017370 | 3.798 |  | |
| HPS1 | Hermansky-Pudlak syndrome 1 | NM_019424 | 3.208 |  | |
| HPS4 | Hermansky-Pudlak syndrome 4 | AI429349 | * |  | |
| HRASLS | HRAS-like suppressor | AI427922 | * |  | |
| HSP90B1 | heat shock protein 90kDa beta (Grp94), member 1 | AI852541 | * |  | |
| HSPA1A | heat shock 70kDa protein 1A | NM_010479 | 3.194 |  | |
| HSPB8 | heat shock 22kDa protein 8 | AI326838 | * |  | |
| IBRDC2 | IBR domain containing 2 | AK038964 | 4.556 |  | |
| IER2 | immediate early response 2 | NM_010499 | 3.32 |  | |
| IFNGR2 | interferon gamma receptor 2 (interferon gamma transducer 1) | AI844547 | * |  | |
| IGFBP3 | insulin-like growth factor binding protein 3 | AI842277 | * |  | |
| IGFBP4 | insulin-like growth factor binding protein 4 | AI849210 | * |  | |
| IGH | immunoglobulin heavy chain complex | BC004786 | 5.99 |  | |
| IL13RA1 | interleukin 13 receptor, alpha 1 | NM_133990 | 3.473 |  | |
| IMPDH1 | IMP (inosine monophosphate) dehydrogenase 1 | NM_011829 | 3.001 |  | |
| INCENP | inner centromere protein antigens 135/155kDa | AI662602 | * |  | |
| INSIG1 | insulin induced gene 1 | AI840465 | * |  | |
| IPO7 | importin 7 | AK048477 | 3.752 |  | |
| IPO8 | importin 8 | AI427065 | * |  | |
| IRF3 | interferon regulatory factor 3 | AI842684 | * |  | |
| ISYNA1 | myo-inositol 1-phosphate synthase A1 | AI465189 | * |  | |
| ITGA3 | integrin, alpha 3 (antigen CD49C, alpha 3 subunit of VLA-3 receptor) | AI447805 | * |  | |
| ITGA4 | integrin, alpha 4 (antigen CD49D, alpha 4 subunit of VLA-4 receptor) | NM_010576 | 5.137 |  | |
| ITGA8 (includes EG:8516) | integrin, alpha 8 | AI447669 | * |  | |
| ITGAE | integrin, alpha E (antigen CD103, human mucosal lymphocyte antigen 1; alpha polypeptide) | AI323739 | * |  | |
| ITGAX | integrin, alpha X (complement component 3 receptor 4 subunit) | NM_021334 | 13.243 |  | |
| ITGB1BP3 | integrin beta 1 binding protein 3 | AK137483 | 4.167 |  | |
| ITPA | inosine triphosphatase (nucleoside triphosphate pyrophosphatase) | AI841281 | * |  | |
| JARID1D | jumonji, AT rich interactive domain 1D | NM_011419 | 20.734 |  | |
| JMY | junction-mediating and regulatory protein | AI426675 | * |  | |
| JUNB | jun B proto-oncogene | NM_008416 | 5.384 |  | |
| KCNC3 | potassium voltage-gated channel, Shaw-related subfamily, member 3 | NM_008422 | 5.014 |  | |
| KCNE1L | KCNE1-like | NM_021487 | 3.54 |  | |
| KCNJ15 | potassium inwardly-rectifying channel, subfamily J, member 15 | AI452066 | * |  | |
| KCNN4 | potassium intermediate/small conductance calcium-activated channel, subfamily N, member 4 | NM_008433 | 4.098 |  | |
| KCNQ1OT1 | KCNQ1 overlapping transcript 1 | NR_001461 | 3.769 |  | |
| KCTD3 | potassium channel tetramerisation domain containing 3 | AI846626 | * |  | |
| KIAA0143 | KIAA0143 protein | AI849823 | * |  | |
| KIAA1267 | KIAA1267 | BC053389 | 3.147 |  | |
| KIF15 | kinesin family member 15 | AI449364 | * |  | |
| KIF17 | kinesin family member 17 | AI427884 | * |  | |
| KIF3A | kinesin family member 3A | AI429298 | * |  | |
| KIF5C | kinesin family member 5C | NM_008449 | 7.593 |  | |
| KLF16 | Kruppel-like factor 16 | NM_078477 | 3.279 |  | |
| KLF5 | Kruppel-like factor 5 (intestinal) | BC006646 | 9.202 |  | |
| KLF6 | Kruppel-like factor 6 | AI448727 | * |  | |
| KLHDC2 | kelch domain containing 2 | AK078408 | 3.244 |  | |
| KLK1B16 | kallikrein 1-related peptidase b16 | NM_008454 | 4.931 |  | |
| KREMEN1 | kringle containing transmembrane protein 1 | AK081707 | 3.075 |  | |
| KRT10 | keratin 10 (epidermolytic hyperkeratosis; keratosis palmaris et plantaris) | AI325191 | * |  | |
| KRT18 | keratin 18 | NM_010664 | 5.275 |  | |
| KRT7 | keratin 7 | AK137217 | 4.308 |  | |
| KRT8 | keratin 8 | NM_031170 | 3.592 |  | |
| KRTCAP2 | keratinocyte associated protein 2 | AI428276 | * |  | |
| LAIR1 | leukocyte-associated immunoglobulin-like receptor 1 | NM_178611 | 5.558 |  | |
| LAMC1 | laminin, gamma 1 (formerly LAMB2) | AI850162 | * |  | |
| LAMP1 | lysosomal-associated membrane protein 1 | AI661788 | * |  | |
| LANCL1 | LanC lantibiotic synthetase component C-like 1 (bacterial) | AI835307 | * |  | |
| LBR | lamin B receptor | AI464404 | * |  | |
| LCAT | lecithin-cholesterol acyltransferase | NM_008490 | 3.233 |  | |
| LDB2 | LIM domain binding 2 | AI853861 | * |  | |
| LDLR | low density lipoprotein receptor (familial hypercholesterolemia) | BC053041 | 3.091 |  | |
| LEFTY1 | left-right determination factor 1 | AI893459 | * |  | |
| LGALS1 | lectin, galactoside-binding, soluble, 1 (galectin 1) | NM_008495 | 3.998 |  | |
| LGALS3 | lectin, galactoside-binding, soluble, 3 | NM_010705 | 5.348 |  | |
| LGP2 | likely ortholog of mouse D11lgp2 | NM_030150 | 3.271 |  | |
| LINGO1 | leucine rich repeat and Ig domain containing 1 | NM_181074 | 13.67 |  | |
| LMNA | lamin A/C | NM_019390 | 3.424 |  | |
| LMO3 | LIM domain only 3 (rhombotin-like 2) | AI854781 | * |  | |
| LOC92345 | hypothetical protein BC008207 | AI661433 | * |  | |
| LPIN3 | lipin 3 | NM_022883 | 3.106 |  | |
| LPXN | leupaxin | NM_134152 | 3.853 |  | |
| LRRFIP1 | leucine rich repeat (in FLII) interacting protein 1 | AI428119 | * |  | |
| M6PRBP1 | mannose-6-phosphate receptor binding protein 1 | AI841293 | * |  | |
| MAGED1 | melanoma antigen family D, 1 | AI848445 | * |  | |
| MAL | mal, T-cell differentiation protein | AI839913 | * |  | |
| MAN1A2 | mannosidase, alpha, class 1A, member 2 | AI451925 | * |  | |
| MAN2A2 | mannosidase, alpha, class 2A, member 2 | NM_172903 | 5.138 |  | |
| MANBA | mannosidase, beta A, lysosomal | AI854873 | * |  | |
| MAP1A | microtubule-associated protein 1A | AK018185 | 11.503 |  | |
| MAP4K1 | mitogen-activated protein kinase kinase kinase kinase 1 | NM_008279 | 4.783 |  | |
| MCM10 | minichromosome maintenance complex component 10 | NM_027290 | 3.13 |  | |
| MCM4 | minichromosome maintenance complex component 4 | AI325074 | * |  | |
| MDFI (includes EG:4188) | MyoD family inhibitor | NM_010783 | 6.024 |  | |
| MDM2 (includes EG:4193) | Mdm2, transformed 3T3 cell double minute 2, p53 binding protein (mouse) | NM_010786 | 6 |  | |
| MERTK | c-mer proto-oncogene tyrosine kinase | AI528587 | * |  | |
| METAP2 | methionyl aminopeptidase 2 | AI848908 | * |  | |
| METRN | meteorin, glial cell differentiation regulator | AI848066 | * |  | |
| MGAT4B | mannosyl (alpha-1,3-)-glycoprotein beta-1,4-N-acetylglucosaminyltransferase, isozyme B | BC026638 | 4.074 |  | |
| MGP | matrix Gla protein | AI427605 | * |  | |
| MIF4GD | MIF4G domain containing | AI449776 | * |  | |
| MIS12 | MIS12, MIND kinetochore complex component, homolog (yeast) | AI465375 | * |  | |
| MKI67 | antigen identified by monoclonal antibody Ki-67 | X82786 | 3.479 |  | |
| MKLN1 | muskelin 1, intracellular mediator containing kelch motifs | AK037967 | 3.651 |  | |
| MKRN1 | makorin, ring finger protein, 1 | AI847663 | * |  | |
| MMP3 | matrix metallopeptidase 3 (stromelysin 1, progelatinase) | NM_010809 | 10.685 |  | |
| MPG | N-methylpurine-DNA glycosylase | AI851936 | * |  | |
| MPND | MPN domain containing | AI848963 | * |  | |
| M-RIP | myosin phosphatase-Rho interacting protein | AI428031 | * |  | |
| MSLN | mesothelin | NM_018857 | 30.074 |  | |
| MT1E | metallothionein 1E | NM_013602 | 3.967 |  | |
| MT2A | metallothionein 2A | NM_008630 | 4.846 |  | |
| MT3 | metallothionein 3 | NM_013603 | 12.516 |  | |
| MT4 | metallothionein 4 | NM_008631 | 31.712 |  | |
| MTMR7 | myotubularin related protein 7 | AI327236 | * |  | |
| MTSS1 | metastasis suppressor 1 | AK046628 | 3.646 |  | |
| MX1 | myxovirus (influenza virus) resistance 1, interferon-inducible protein p78 (mouse) | NM_013606 | 4.658 |  | |
| MXD4 | MAX dimerization protein 4 | AI849076 | * |  | |
| MYL4 | myosin, light chain 4, alkali; atrial, embryonic | NM_010858 | 5.287 |  | |
| MYLK | myosin, light chain kinase | AI429407 | * |  | |
| MYO5A | myosin VA (heavy chain 12, myoxin) | NM_010864 | 6.34 |  | |
| MYOG | myogenin (myogenic factor 4) | NM_031189 | 3.872 |  | |
| NAB2 | NGFI-A binding protein 2 (EGR1 binding protein 2) | NM_008668 | 5.201 |  | |
| NAPB | N-ethylmaleimide-sensitive factor attachment protein, beta | X61450 | 7.685 |  | |
| NARS2 | asparaginyl-tRNA synthetase 2, mitochondrial (putative) | AK081401 | 3.356 |  | |
| NCAM1 | neural cell adhesion molecule 1 | X14526 | 11.604 |  | |
| NCAN | neurocan | NM_007789 | 4.938 |  | |
| NCF4 | neutrophil cytosolic factor 4, 40kDa | NM_008677 | 3.411 |  | |
| NCOA7 | nuclear receptor coactivator 7 | NM_172495 | 3.851 |  | |
| NDC80 | NDC80 homolog, kinetochore complex component (S. cerevisiae) | AI450066 | * |  | |
| NDFIP1 | Nedd4 family interacting protein 1 | AI837293 | * |  | |
| NDN | necdin homolog (mouse) | AI853718 | * |  | |
| NEB | nebulin | AK086142 | 3.2 |  | |
| NECAP1 | NECAP endocytosis associated 1 | AI842479 | * |  | |
| NEFL | neurofilament, light polypeptide 68kDa | AI835609 | * |  | |
| NES | nestin | NM_016701 | 4.24 |  | |
| NFATC2 | nuclear factor of activated T-cells, cytoplasmic, calcineurin-dependent 2 | AK081853 | 5.746 |  | |
| NFKB1 | nuclear factor of kappa light polypeptide gene enhancer in B-cells 1 (p105) | BC050841 | 3.748 |  | |
| NFKB2 | nuclear factor of kappa light polypeptide gene enhancer in B-cells 2 (p49/p100) | NM_019408 | 3.66 |  | |
| NISCH | nischarin | AK088377 | 4.067 |  | |
| NKTR | natural killer-tumor recognition sequence | NM_010918 | 3.306 |  | |
| NMT2 | N-myristoyltransferase 2 | AK049483 | 3.136 |  | |
| N-PAC | cytokine-like nuclear factor n-pac | AK037868 | 3.297 |  | |
| NPNT | nephronectin | NM_001029836 | 3.83 |  | |
| NPTX1 | neuronal pentraxin I | AI854896 | * |  | |
| NPY | neuropeptide Y | AI848386 | * |  | |
| NRAS | neuroblastoma RAS viral (v-ras) oncogene homolog | NM_010937 | 3.539 |  | |
| NRF1 | nuclear respiratory factor 1 | AK143596 | 3.224 |  | |
| NSFL1C | NSFL1 (p97) cofactor (p47) | AI835943 | * |  | |
| NT5E | 5'-nucleotidase, ecto (CD73) | AI447772 | * |  | |
| NUDT14 | nudix (nucleoside diphosphate linked moiety X)-type motif 14 | AI426677 | * |  | |
| NUP98 | nucleoporin 98kDa | AK030935 | 3.196 |  | |
| NXN | nucleoredoxin | NM_008750 | 3.325 |  | |
| OAS2 | 2'-5'-oligoadenylate synthetase 2, 69/71kDa | NM_145227 | 5.474 |  | |
| OASL | 2'-5'-oligoadenylate synthetase-like | NM_145209 | 3.476 |  | |
| ODC1 | ornithine decarboxylase 1 | NM_013614 | 3.382 |  | |
| ODF2 | outer dense fiber of sperm tails 2 | AI449749 | * |  | |
| OGDH | oxoglutarate (alpha-ketoglutarate) dehydrogenase (lipoamide) | AI325471 | * |  | |
| OGFR | opioid growth factor receptor | AI464566 | * |  | |
| OPA3 | optic atrophy 3 (autosomal recessive, with chorea and spastic paraplegia) | AI852078 | * |  | |
| ORC3L | origin recognition complex, subunit 3-like (yeast) | AI427116 | * |  | |
| P2RY6 | pyrimidinergic receptor P2Y, G-protein coupled, 6 | NM_183168 | 3.815 |  | |
| P4HB | procollagen-proline, 2-oxoglutarate 4-dioxygenase (proline 4-hydroxylase), beta polypeptide | AI836363 | * |  | |
| P8 | nuclear protein 1 | AI852641 | * |  | |
| PANK1 | pantothenate kinase 1 | AI644488 | * |  | |
| PANK2 | pantothenate kinase 2 (Hallervorden-Spatz syndrome) | AI850976 | * |  | |
| PAPPA | pregnancy-associated plasma protein A, pappalysin 1 | AI427027 | * |  | |
| PARVA | parvin, alpha | AI448149 | * |  | |
| PBX1 | pre-B-cell leukemia homeobox 1 | AK144446 | 3.051 |  | |
| PCDH7 | protocadherin 7 | AI449381 | * |  | |
| PCF11 | PCF11, cleavage and polyadenylation factor subunit, homolog (S. cerevisiae) | BC048838 | 3.576 |  | |
| PCID1 | PCI domain containing 1 (herpesvirus entry mediator) | AI449207 | * |  | |
| PCMT1 | protein-L-isoaspartate (D-aspartate) O-methyltransferase | AI841469 | * |  | |
| PCTK2 | PCTAIRE protein kinase 2 | AK039890 | 3.932 |  | |
| PDE1C | phosphodiesterase 1C, calmodulin-dependent 70kDa | AI845662 | * |  | |
| PDE4B | phosphodiesterase 4B, cAMP-specific (phosphodiesterase E4 dunce homolog, Drosophila) | AK171700 | 3.53 |  | |
| PDGFB | platelet-derived growth factor beta polypeptide (simian sarcoma viral (v-sis) oncogene homolog) | NM_011057 | 4.074 |  | |
| PDHA1 (includes EG:5160) | pyruvate dehydrogenase (lipoamide) alpha 1 | AI528531 | * |  | |
| PDIA6 | protein disulfide isomerase family A, member 6 | AK148928 | 6.013 |  | |
| PDLIM7 | PDZ and LIM domain 7 (enigma) | AI836896 | * |  | |
| PDZK1IP1 | PDZK1 interacting protein 1 | AI449816 | * |  | |
| PEBP4 | phosphatidylethanolamine-binding protein 4 | AI429082 | * |  | |
| PEG3 (includes EG:5178) | paternally expressed 3 | NM_008817 | 4.01 |  | |
| PERP (includes EG:64065) | PERP, TP53 apoptosis effector | NM_022032 | 5.5 |  | |
| PEX1 | peroxisome biogenesis factor 1 | AK082305 | 3.872 |  | |
| PFKFB3 | 6-phosphofructo-2-kinase/fructose-2,6-biphosphatase 3 | AI326331 | * |  | |
| PHF21A | PHD finger protein 21A | AK083682 | 4.01 |  | |
| PHKA2 | phosphorylase kinase, alpha 2 (liver) | AK045618 | 3.526 |  | |
| PHKG2 | phosphorylase kinase, gamma 2 (testis) | AK089062 | 3.175 |  | |
| PHLDA1 | pleckstrin homology-like domain, family A, member 1 | NM_009344 | 7.141 |  | |
| PIGK | phosphatidylinositol glycan anchor biosynthesis, class K | AI852352 | * |  | |
| PIGO | phosphatidylinositol glycan anchor biosynthesis, class O | AI850968 | * |  | |
| PIK3R5 | phosphoinositide-3-kinase, regulatory subunit 5, p101 | NM_177320 | 3.882 |  | |
| PIP5K1A | phosphatidylinositol-4-phosphate 5-kinase, type I, alpha | AI851569 | * |  | |
| PIP5K1C | phosphatidylinositol-4-phosphate 5-kinase, type I, gamma | AI835305 | * |  | |
| PISD | phosphatidylserine decarboxylase | AI449740 | * |  | |
| PITX2 | paired-like homeodomain transcription factor 2 | AK035083 | 3.266 |  | |
| PKP3 | plakophilin 3 | NM_019762 | 3.362 |  | |
| PLAC8 | placenta-specific 8 | NM_139198 | 4.621 |  | |
| PLEKHA5 | pleckstrin homology domain containing, family A member 5 | AI428202 | * |  | |
| PLEKHO1 | pleckstrin homology domain containing, family O member 1 | NM_023320 | 4.133 |  | |
| PLK2 | polo-like kinase 2 (Drosophila) | NM_152804 | 4.099 |  | |
| PMAIP1 | phorbol-12-myristate-13-acetate-induced protein 1 | NM_021451 | 4.835 |  | |
| POLE3 | polymerase (DNA directed), epsilon 3 (p17 subunit) | AI326325 | * |  | |
| POLR1A | polymerase (RNA) I polypeptide A, 194kDa | AK087773 | 3.712 |  | |
| POLR3C | polymerase (RNA) III (DNA directed) polypeptide C (62kD) | AI840272 | * |  | |
| POLR3G | polymerase (RNA) III (DNA directed) polypeptide G (32kD) | BC066818 | 3.095 |  | |
| POLRMT | polymerase (RNA) mitochondrial (DNA directed) | AI836334 | * |  | |
| POMC | proopiomelanocortin (adrenocorticotropin/ beta-lipotropin/ alpha-melanocyte stimulating hormone/ beta-melanocyte stimulating hormone/ beta-endorphin) | NM_008895 | 3.218 |  | |
| PPARGC1B | peroxisome proliferator-activated receptor gamma, coactivator 1 beta | AK042378 | 3.372 |  | |
| PPIG | peptidylprolyl isomerase G (cyclophilin G) | AI854234 | * |  | |
| PPIH | peptidylprolyl isomerase H (cyclophilin H) | AI844569 | * |  | |
| PPP1R13B | protein phosphatase 1, regulatory (inhibitor) subunit 13B | AI854233 | * |  | |
| PPP1R14B | protein phosphatase 1, regulatory (inhibitor) subunit 14B | NM_008889 | 3.393 |  | |
| PPP1R3D | protein phosphatase 1, regulatory (inhibitor) subunit 3D | AI851283 | * |  | |
| PPP2R1A | protein phosphatase 2 (formerly 2A), regulatory subunit A , alpha isoform | AI842022 | * |  | |
| PPP2R3A (includes EG:5523) | protein phosphatase 2 (formerly 2A), regulatory subunit B'', alpha | XM_001004721 | 4.605 |  | |
| PPP3CC | protein phosphatase 3 (formerly 2B), catalytic subunit, gamma isoform | AI323727 | * |  | |
| PRC1 | protein regulator of cytokinesis 1 | NM_145150 | 3.089 |  | |
| PRDX3 | peroxiredoxin 3 | AI836312 | * |  | |
| PREP | prolyl endopeptidase | AI429335 | * |  | |
| PRKAA1 | protein kinase, AMP-activated, alpha 1 catalytic subunit | NM_001013367 | 3.118 |  | |
| PRKAB2 | protein kinase, AMP-activated, beta 2 non-catalytic subunit | AI427023 | * |  | |
| PRKAG2 | protein kinase, AMP-activated, gamma 2 non-catalytic subunit | NM_145401 | 3.131 |  | |
| PRKCD | protein kinase C, delta | NM_011103 | 3.034 |  | |
| PRKDC | protein kinase, DNA-activated, catalytic polypeptide | AI893647 | * |  | |
| PRKG2 | protein kinase, cGMP-dependent, type II | AI447755 | * |  | |
| PRM2 | protamine 2 | AI528784 | * |  | |
| PRND | prion protein 2 (dublet) | AI428337 | * |  | |
| PRNP | prion protein (p27-30) (Creutzfeldt-Jakob disease, Gerstmann-Strausler-Scheinker syndrome, fatal familial insomnia) | NM_011170 | 8.236 |  | |
| PRSS23 | protease, serine, 23 | NM_029614 | 3.17 |  | |
| PSCD2 | pleckstrin homology, Sec7 and coiled-coil domains 2 (cytohesin-2) | AI835125 | * |  | |
| PSCD4 | pleckstrin homology, Sec7 and coiled-coil domains 4 | NM_028195 | 3.895 |  | |
| PSMD2 | proteasome (prosome, macropain) 26S subunit, non-ATPase, 2 | AI414985 | * |  | |
| PSMD4 | proteasome (prosome, macropain) 26S subunit, non-ATPase, 4 | AB029144 | 3.371 |  | |
| PSMD7 | proteasome (prosome, macropain) 26S subunit, non-ATPase, 7 (Mov34 homolog) | AK012463 | 3.241 |  | |
| PSTPIP1 | proline-serine-threonine phosphatase interacting protein 1 | NM_011193 | 5.825 |  | |
| PTPRC | protein tyrosine phosphatase, receptor type, C | NM_011210 | 3.342 |  | |
| PURA | purine-rich element binding protein A | AI836331 | * |  | |
| PURB | purine-rich element binding protein B | AI850922 | * |  | |
| PUS1 | pseudouridylate synthase 1 | AI429184 | * |  | |
| PVR | poliovirus receptor | NM_027514 | 4.941 |  | |
| PVRL1 | poliovirus receptor-related 1 (herpesvirus entry mediator C; nectin) | AI836941 | * |  | |
| RAB14 | RAB14, member RAS oncogene family | AK083451 | 3.529 |  | |
| RAB3GAP2 | RAB3 GTPase activating protein subunit 2 (non-catalytic) | AI449737 | * |  | |
| RAB40C | RAB40C, member RAS oncogene family | AI429229 | * |  | |
| RAB6A | RAB6A, member RAS oncogene family | AI852932 | * |  | |
| RAB8B | RAB8B, member RAS oncogene family | NM_173413 | 3.324 |  | |
| RANBP1 | RAN binding protein 1 | AI836209 | * |  | |
| RAP2A | RAP2A, member of RAS oncogene family | AK018024 | 3.832 |  | |
| RAPGEF5 | Rap guanine nucleotide exchange factor (GEF) 5 | AI327232 | * |  | |
| RASSF4 | Ras association (RalGDS/AF-6) domain family 4 | NM_178045 | 3.733 |  | |
| RB1 | retinoblastoma 1 (including osteosarcoma) | AI847188 | * |  | |
| RBM10 | RNA binding motif protein 10 | AI854477 | * |  | |
| RCOR3 | REST corepressor 3 | AI464449 | * |  | |
| RCP9 | calcitonin gene-related peptide-receptor component protein | AI843531 | * |  | |
| RDH11 | retinol dehydrogenase 11 (all-trans/9-cis/11-cis) | AI450559 | * |  | |
| RGS12 | regulator of G-protein signalling 12 | AI450971 | * |  | |
| RGS2 | regulator of G-protein signalling 2, 24kDa | AI464441 | * |  | |
| RHOA | ras homolog gene family, member A | AI846668 | * |  | |
| RHOC | ras homolog gene family, member C | NM_007484 | 3.127 |  | |
| RHOT1 | ras homolog gene family, member T1 | AI427785 | * |  | |
| RHPN2 | rhophilin, Rho GTPase binding protein 2 | AI851792 | * |  | |
| RIN1 | Ras and Rab interactor 1 | NM_145495 | 7.845 |  | |
| RINT-1 | RAD50 interactor 1 | AK005284 | 3.904 |  | |
| RIOK1 | RIO kinase 1 (yeast) | AK079540 | 3.002 |  | |
| RMRP | RNA component of mitochondrial RNA processing endoribonuclease | NR_001460 | 4.734 |  | |
| RNASEH1 | ribonuclease H1 | AI852889 | * |  | |
| RNF111 | ring finger protein 111 | NM_033604 | 3.488 |  | |
| ROPN1L | ropporin 1-like | NM_145852 | 3.172 |  | |
| RPL10 | ribosomal protein L10 | AI836475 | * |  | |
| RPL5 | ribosomal protein L5 | AI835381 | * |  | |
| RPN1 | ribophorin I | AI846708 | * |  | |
| RPS6KA5 (includes EG:9252) | ribosomal protein S6 kinase, 90kDa, polypeptide 5 | AI842520 | * |  | |
| RPS6KB1 | ribosomal protein S6 kinase, 70kDa, polypeptide 1 | AI838154 | * |  | |
| RRAD | Ras-related associated with diabetes | NM_019662 | 21.618 |  | |
| RRBP1 | ribosome binding protein 1 homolog 180kDa (dog) | AK050411 | 4.437 |  | |
| RUFY1 | RUN and FYVE domain containing 1 | AI465156 | * |  | |
| RUFY2 | RUN and FYVE domain containing 2 | AI852705 | * |  | |
| RUNX1 | runt-related transcription factor 1 (acute myeloid leukemia 1; aml1 oncogene) | NM_009821 | 8.385 |  | |
| RYR3 | ryanodine receptor 3 | XM_619795 | 3.126 |  | |
| S100A13 | S100 calcium binding protein A13 | NM_009113 | 4.98 |  | |
| S100A4 | S100 calcium binding protein A4 | NM_011311 | 11.07 |  | |
| SACM1L | SAC1 suppressor of actin mutations 1-like (yeast) | AK036777 | 3.055 |  | |
| SAMD4A | sterile alpha motif domain containing 4A | AK018954 | 3.754 |  | |
| SAPS3 | SAPS domain family, member 3 | AK018652 | 3.524 |  | |
| SBDS | Shwachman-Bodian-Diamond syndrome | AI836084 | * |  | |
| SBNO2 | strawberry notch homolog 2 (Drosophila) | NM_183426 | 3.596 |  | |
| SCGN | secretagogin, EF-hand calcium binding protein | AI849182 | * |  | |
| SCHIP1 | schwannomin interacting protein 1 | AI449887 | * |  | |
| SCLY | selenocysteine lyase | AK036533 | 3.817 |  | |
| SCXA | scleraxis homolog A (mouse) | NM_198885 | 4.77 |  | |
| SEH1L | SEH1-like (S. cerevisiae) | AI428383 | * |  | |
| SELM | selenoprotein M | AI839954 | * |  | |
| SEMA6A | sema domain, transmembrane domain (TM), and cytoplasmic domain, (semaphorin) 6A | AI851372 | * |  | |
| SEPHS1 | selenophosphate synthetase 1 | AI849401 | * |  | |
| SERINC1 | serine incorporator 1 | AI854679 | * |  | |
| SERINC2 | serine incorporator 2 | NM_172702 | 3.086 |  | |
| SERPINB1 | serpin peptidase inhibitor, clade B (ovalbumin), member 1 | NM_025429 | 8.552 |  | |
| SERPINB1B | serine (or cysteine) peptidase inhibitor, clade B, member 1b | NM_173052 | 4.137 |  | |
| SF3A1 | splicing factor 3a, subunit 1, 120kDa | AI854209 | * |  | |
| SFRS11 | splicing factor, arginine/serine-rich 11 | AI843578 | * |  | |
| SFRS2 | splicing factor, arginine/serine-rich 2 | AI837001 | * |  | |
| SGMS2 | sphingomyelin synthase 2 | NM_028943 | 4.845 |  | |
| SGPP1 | sphingosine-1-phosphate phosphatase 1 | AI835784 | * |  | |
| SH3BP2 | SH3-domain binding protein 2 | NM_011893 | 5.017 |  | |
| SIN3A | SIN3 homolog A, transcription regulator (yeast) | AI426974 | * |  | |
| SIRT3 | sirtuin (silent mating type information regulation 2 homolog) 3 (S. cerevisiae) | AI464384 | * |  | |
| SLC15A2 | solute carrier family 15 (H+/peptide transporter), member 2 | AI846682 | * |  | |
| SLC20A1 | solute carrier family 20 (phosphate transporter), member 1 | AI385598 | * |  | |
| SLC25A39 | solute carrier family 25, member 39 | AI839261 | * |  | |
| SLC25A5 | solute carrier family 25 (mitochondrial carrier; adenine nucleotide translocator), member 5 | AI325068 | * |  | |
| SLC2A6 | solute carrier family 2 (facilitated glucose transporter), member 6 | NM_172659 | 4.099 |  | |
| SLC32A1 | solute carrier family 32 (GABA vesicular transporter), member 1 | AI836076 | * |  | |
| SLC35B1 | solute carrier family 35, member B1 | AI851443 | * |  | |
| SLC37A4 | solute carrier family 37 (glucose-6-phosphate transporter), member 4 | AI845186 | * |  | |
| SLC39A5 | solute carrier family 39 (metal ion transporter), member 5 | AI465146 | * |  | |
| SLC3A2 | solute carrier family 3 (activators of dibasic and neutral amino acid transport), member 2 | AI840451 | * |  | |
| SLC4A3 | solute carrier family 4, anion exchanger, member 3 | AK161240 | 4.97 |  | |
| SLC4A4 | solute carrier family 4, sodium bicarbonate cotransporter, member 4 | AI465149 | * |  | |
| SLC7A11 | solute carrier family 7, (cationic amino acid transporter, y+ system) member 11 | AI451155 | * |  | |
| SLN (includes EG:6588) | sarcolipin | NM_025540 | 8.421 |  | |
| SMARCA2 | SWI/SNF related, matrix associated, actin dependent regulator of chromatin, subfamily a, member 2 | AI839352 | * |  | |
| SMARCB1 | SWI/SNF related, matrix associated, actin dependent regulator of chromatin, subfamily b, member 1 | AI835308 | * |  | |
| SMARCE1 | SWI/SNF related, matrix associated, actin dependent regulator of chromatin, subfamily e, member 1 | AI852152 | * |  | |
| SMC3 | structural maintenance of chromosomes 3 | AI844164 | * |  | |
| SMG6 | Smg-6 homolog, nonsense mediated mRNA decay factor (C. elegans) | BC006644 | 3.701 |  | |
| SMG7 | Smg-7 homolog, nonsense mediated mRNA decay factor (C. elegans) | AI853422 | * |  | |
| SMOX | spermine oxidase | NM_145533 | 3.734 |  | |
| SMS | spermine synthase | AK049780 | 4.438 |  | |
| SMUG1 | single-strand-selective monofunctional uracil-DNA glycosylase 1 | AK045633 | 3.58 |  | |
| SNCA | synuclein, alpha (non A4 component of amyloid precursor) | AI844674 | * |  | |
| SNF8 | SNF8, ESCRT-II complex subunit, homolog (S. cerevisiae) | BC030490 | 3.426 |  | |
| SNRPA | small nuclear ribonucleoprotein polypeptide A | AI324697 | * |  | |
| SNRPA1 | small nuclear ribonucleoprotein polypeptide A' | AI465196 | * |  | |
| SNRPB | small nuclear ribonucleoprotein polypeptides B and B1 | AI840565 | * |  | |
| SNX5 | sorting nexin 5 | AK088223 | 3.209 |  | |
| SOAT1 | sterol O-acyltransferase (acyl-Coenzyme A: cholesterol acyltransferase) 1 | NM_009230 | 3.628 |  | |
| SOCS2 | suppressor of cytokine signaling 2 | AI464459 | * |  | |
| SOCS3 | suppressor of cytokine signaling 3 | NM_007707 | 5.37 |  | |
| SOCS5 | suppressor of cytokine signaling 5 | AI447636 | * |  | |
| SOCS7 | suppressor of cytokine signaling 7 | NM_138657 | 3.916 |  | |
| SORD | sorbitol dehydrogenase | AI845824 | * |  | |
| SOX10 | SRY (sex determining region Y)-box 10 | AI847435 | * |  | |
| SOX11 | SRY (sex determining region Y)-box 11 | AF009414 | 23.312 |  | |
| SOX9 | SRY (sex determining region Y)-box 9 (campomelic dysplasia, autosomal sex-reversal) | NM_011448 | 3.408 |  | |
| SPAG1 | sperm associated antigen 1 | NM_012031 | 3.354 |  | |
| SPAG5 | sperm associated antigen 5 | NM_017407 | 4.447 |  | |
| SPAG9 | sperm associated antigen 9 | AK014060 | 3.019 |  | |
| SPAST | spastin | AI853690 | * |  | |
| SPC25 | SPC25, NDC80 kinetochore complex component, homolog (S. cerevisiae) | AI447803 | * |  | |
| SPOCK2 | sparc/osteonectin, cwcv and kazal-like domains proteoglycan (testican) 2 | NM_052994 | 12.76 |  | |
| SPRED2 | sprouty-related, EVH1 domain containing 2 | AK040092 | 3.16 |  | |
| SPSB2 | splA/ryanodine receptor domain and SOCS box containing 2 | NM_013539 | 7.425 |  | |
| SPZ1 | spermatogenic leucine zipper 1 | AI429130 | * |  | |
| SRGAP3 | SLIT-ROBO Rho GTPase activating protein 3 | AI451149 | * |  | |
| SRM | spermidine synthase | AI843751 | * |  | |
| SRP9 | signal recognition particle 9kDa | AI835924 | * |  | |
| SRRM2 | serine/arginine repetitive matrix 2 | AI447673 | * |  | |
| SSR4 | signal sequence receptor, delta (translocon-associated protein delta) | AI847951 | * |  | |
| SSTR2 | somatostatin receptor 2 | NM_009217 | 3.379 |  | |
| ST6GALNAC4 | ST6 (alpha-N-acetyl-neuraminyl-2,3-beta-galactosyl-1,3)-N-acetylgalactosaminide alpha-2,6-sialyltransferase 4 | NM_011373 | 3.914 |  | |
| ST6GALNAC6 | ST6 (alpha-N-acetyl-neuraminyl-2,3-beta-galactosyl-1,3)-N-acetylgalactosaminide alpha-2,6-sialyltransferase 6 | AI852309 | * |  | |
| STAT3 | signal transducer and activator of transcription 3 (acute-phase response factor) | AI837104 | * |  | |
| STK16 | serine/threonine kinase 16 | AI835611 | * |  | |
| STK36 | serine/threonine kinase 36, fused homolog (Drosophila) | NM_175031 | 3.276 |  | |
| STK39 | serine threonine kinase 39 (STE20/SPS1 homolog, yeast) | AI854240 | * |  | |
| STMN3 | stathmin-like 3 | AI841267 | * |  | |
| STOM | stomatin | NM_013515 | 4.92 |  | |
| STS-1 | Cbl-interacting protein Sts-1 | NM_176860 | 7.582 |  | |
| SULT2B1 | sulfotransferase family, cytosolic, 2B, member 1 | AI851328 | * |  | |
| SUPT3H | suppressor of Ty 3 homolog (S. cerevisiae) | AI429135 | * |  | |
| SUPT6H | suppressor of Ty 6 homolog (S. cerevisiae) | AI854263 | * |  | |
| SURF4 | surfeit 4 | AI842147 | * |  | |
| SUV39H2 | suppressor of variegation 3-9 homolog 2 (Drosophila) | AI426565 | * |  | |
| SUV420H1 | suppressor of variegation 4-20 homolog 1 (Drosophila) | AI465201 | * |  | |
| SV2A | synaptic vesicle glycoprotein 2A | AI835486 | * |  | |
| SVIL | supervillin | NM_178046 | 3.621 |  | |
| SYK | spleen tyrosine kinase | NM_011518 | 3.132 |  | |
| SYMPK | symplekin | AI449890 | * |  | |
| SYNJ1 | synaptojanin 1 | AI426972 | * |  | |
| SYPL2 | synaptophysin-like 2 | AI427574 | * |  | |
| SYT12 | synaptotagmin XII | NM_134164 | 4.854 |  | |
| TAC4 | tachykinin 4 (hemokinin) | NM_053093 | 10.733 |  | |
| TACC3 | transforming, acidic coiled-coil containing protein 3 | AI429136 | * |  | |
| TAL2 | T-cell acute lymphocytic leukemia 2 | NM_009317 | 4.63 |  | |
| TAOK3 | TAO kinase 3 | AI894261 | * |  | |
| TAX1BP3 | Tax1 (human T-cell leukemia virus type I) binding protein 3 | AI842665 | * |  | |
| TBX6 | T-box 6 | NM_011538 | 3.091 |  | |
| TCEA2 | transcription elongation factor A (SII), 2 | AI326127 | * |  | |
| TCERG1 | transcription elongation regulator 1 | AI843647 | * |  | |
| TCF23 | transcription factor 23 | NM_053085 | 3.252 |  | |
| TCFL5 | transcription factor-like 5 (basic helix-loop-helix) | AI427864 | * |  | |
| TCIRG1 | T-cell, immune regulator 1, ATPase, H+ transporting, lysosomal V0 subunit A3 | NM_016921 | 3.951 |  | |
| TEAD1 | TEA domain family member 1 (SV40 transcriptional enhancer factor) | AK048761 | 4.989 |  | |
| TEAD4 | TEA domain family member 4 | D87966 | 7.848 |  | |
| TFB1M | transcription factor B1, mitochondrial | AI429207 | * |  | |
| TGIF2 | TGFB-induced factor homeobox 2 | AI427094 | * |  | |
| THPO | thrombopoietin (myeloproliferative leukemia virus oncogene ligand, megakaryocyte growth and development factor) | NM_009379 | 4.181 |  | |
| THSD1 | thrombospondin, type I, domain containing 1 | AI448749 | * |  | |
| TIAM2 | T-cell lymphoma invasion and metastasis 2 | NM_011878 | 5.761 |  | |
| TIMELESS | timeless homolog (Drosophila) | AI426736 | * |  | |
| TIMM17A | translocase of inner mitochondrial membrane 17 homolog A (yeast) | AI839512 | * |  | |
| TIMP1 | TIMP metallopeptidase inhibitor 1 | NM_011593 | 20.475 |  | |
| TMC6 | transmembrane channel-like 6 | NM_145439 | 3.009 |  | |
| TMEM37 | transmembrane protein 37 | AI848779 | * |  | |
| TMEM43 | transmembrane protein 43 | NM_028766 | 3.111 |  | |
| TMEM87A | transmembrane protein 87A | AK037375 | 3.679 |  | |
| TMSB10 | thymosin, beta 10 | NM_025284 | 6.006 |  | |
| TNFRSF12A | tumor necrosis factor receptor superfamily, member 12A | NM_013749 | 4.911 |  | |
| TNIK | TRAF2 and NCK interacting kinase | AK021275 | 3.525 |  | |
| TNIP1 | TNFAIP3 interacting protein 1 | AI427921 | * |  | |
| TNNT2 | troponin T type 2 (cardiac) | NM_011619 | 7.502 |  | |
| TNP1 | transition protein 1 (during histone to protamine replacement) | AI528769 | * |  | |
| TOB1 | transducer of ERBB2, 1 | AI837817 | * |  | |
| TOMM40 | translocase of outer mitochondrial membrane 40 homolog (yeast) | AI845875 | * |  | |
| TOP1 | topoisomerase (DNA) I | AK083930 | 3.331 |  | |
| TOX | thymocyte selection-associated high mobility group box | AI849768 | * |  | |
| TP53INP1 | tumor protein p53 inducible nuclear protein 1 | AI835817 | * |  | |
| TP63 | tumor protein p73-like | NM_011641 | 4.644 |  | |
| TPST1 | tyrosylprotein sulfotransferase 1 | AI413734 | * |  | |
| TRADD | TNFRSF1A-associated via death domain | AI844060 | * |  | |
| TREM2 | triggering receptor expressed on myeloid cells 2 | NM_031254 | 9.698 |  | |
| TRERF1 | transcriptional regulating factor 1 | NM_172622 | 3.318 |  | |
| TRH | thyrotropin-releasing hormone | AI836004 | * |  | |
| TRIAP1 | TP53 regulated inhibitor of apoptosis 1 | AI852179 | * |  | |
| TRIM16 | tripartite motif-containing 16 | AI426590 | * |  | |
| TRIM35 | tripartite motif-containing 35 | AI846067 | * |  | |
| TRIP13 | thyroid hormone receptor interactor 13 | AI852645 | * |  | |
| TROVE2 | TROVE domain family, member 2 | AI834985 | * |  | |
| TRPS1 | trichorhinophalangeal syndrome I | AK036590 | 3.574 |  | |
| TSN | translin | AI426573 | * |  | |
| TSNAX | translin-associated factor X | AI852243 | * |  | |
| TSPAN31 | tetraspanin 31 | AI844321 | * |  | |
| TSPAN4 | tetraspanin 4 | AI839119 | * |  | |
| TTC1 | tetratricopeptide repeat domain 1 | AI849433 | * |  | |
| TTC9 | tetratricopeptide repeat domain 9 | NM_001033149 | 8.231 |  | |
| TWF1 | twinfilin, actin-binding protein, homolog 1 (Drosophila) | AI666631 | * |  | |
| UBE2E2 | ubiquitin-conjugating enzyme E2E 2 (UBC4/5 homolog, yeast) | AK044807 | 3.525 |  | |
| UBE2J2 | ubiquitin-conjugating enzyme E2, J2 (UBC6 homolog, yeast) | AI449873 | * |  | |
| UBP1 | upstream binding protein 1 (LBP-1a) | AI850407 | * |  | |
| UCHL1 | ubiquitin carboxyl-terminal esterase L1 (ubiquitin thiolesterase) | NM_011670 | 4.29 |  | |
| UCK2 (includes EG:7371) | uridine-cytidine kinase 2 | NM_030724 | 4.74 |  | |
| UCN2 | urocortin 2 | AF331517 | 5.281 |  | |
| UCP1 | uncoupling protein 1 (mitochondrial, proton carrier) | NM_009463 | 5.674 |  | |
| UGCG | UDP-glucose ceramide glucosyltransferase | NM_011673 | 3.239 |  | |
| UHRF2 | ubiquitin-like, containing PHD and RING finger domains, 2 | AI429332 | * |  | |
| UNC5B | unc-5 homolog B (C. elegans) | AI835700 | * |  | |
| UQCRC1 | ubiquinol-cytochrome c reductase core protein I | AI835613 | * |  | |
| UROD | uroporphyrinogen decarboxylase | AI854174 | * |  | |
| USP21 | ubiquitin specific peptidase 21 | AI843395 | * |  | |
| USP33 | ubiquitin specific peptidase 33 | AI853456 | * |  | |
| USP36 | ubiquitin specific peptidase 36 | AI851559 | * |  | |
| USP48 | ubiquitin specific peptidase 48 | AI449795 | * |  | |
| VASN | vasorin | NM_139307 | 3.405 |  | |
| VCPIP1 | valosin containing protein (p97)/p47 complex interacting protein 1 | BC052908 | 3.565 |  | |
| VLDLR | very low density lipoprotein receptor | AI666417 | * |  | |
| VNN1 | vanin 1 | AI644483 | * |  | |
| VPS52 | vacuolar protein sorting 52 homolog (S. cerevisiae) | AI848997 | * |  | |
| VPS72 | vacuolar protein sorting 72 homolog (S. cerevisiae) | AI854449 | * |  | |
| WBP4 | WW domain binding protein 4 (formin binding protein 21) | AI845671 | * |  | |
| WBSCR17 | Williams-Beuren syndrome chromosome region 17 | NM_145218 | 4.845 |  | |
| WDFY3 | WD repeat and FYVE domain containing 3 | AK049456 | 3.065 |  | |
| WDR82 | -- | AI852154 | * |  | |
| WIPF3 | WAS/WASL interacting protein family, member 3 | AK078889 | 3.658 |  | |
| XPNPEP1 | X-prolyl aminopeptidase (aminopeptidase P) 1, soluble | AI852589 | * |  | |
| XPO7 | exportin 7 | AI429475 | * |  | |
| XPOT | exportin, tRNA (nuclear export receptor for tRNAs) | AI450562 | * |  | |
| YBX2 | Y box binding protein 2 | AI429205 | * |  | |
| ZDHHC3 | zinc finger, DHHC-type containing 3 | AI465153 | * |  | |
| ZFP91 | zinc finger protein 91 homolog (mouse) | AK084959 | 5.488 |  | |
| ZNF148 | zinc finger protein 148 | AI465377 | * |  | |
| ZNF287 | zinc finger protein 287 | AK031950 | 6.461 |  | |
|  |  |  |  |  | |
|  |  |  |  |  | |
|  |  |  |  |  | |

A fold change value is provided for genes identified on the Agilent arrays at 14 days post Dox treatment. A * denotes that the gene was identified on the BMAP arrays and had a fold change greater than 3 for at least one of the time points tested 0, 4, 7, 14, 30 or 60 days post Dox treatment.
