# Supplementary material for: Activation of p53-regulated pro-apoptotic signaling pathways in PrP-mediated myopathy
Source: BMC Genomics. 2009 Apr 28;10:201. doi: 10.1186/1471-2164-10-201 (PMC2683871; doi:10.1186/1471-2164-10-201)
Supplement: Additional File 2 — Genes down-regulated in Tg(HQK) muscle following induction of PrP over-expression. The data provided represents a list of genes determined to be down-regulated following induction of PrP over-expression in Tg(HQK) muscle. Genes include those found to be temporally de-regulated on the BMAP platform and those found using the Agilent microarray platform at 14 days post induction. [file 1471-2164-10-201-S2.doc]

|  |  |  |  |
| --- | --- | --- | --- |
| **Name** | **Description** | **GenBank** | **Fold Change** |
|  |  |  |  |
| ABAT | 4-aminobutyrate aminotransferase | AI853123 | * |
| ABCB4 | ATP-binding cassette, sub-family B (MDR/TAP), member 4 | NM_008830 | -5.143 |
| ACAD9 | acyl-Coenzyme A dehydrogenase family, member 9 | AI846542 | * |
| ACADSB | acyl-Coenzyme A dehydrogenase, short/branched chain | NM_025826 | -3.582 |
| ACTC1 | actin, alpha, cardiac muscle 1 | NM_009608 | -9.359 |
| ACTG2 | actin, gamma 2, smooth muscle, enteric | NM_009610 | -6.081 |
| ACY3 | aspartoacylase (aminocyclase) 3 | AI325516 | * |
| ACYP1 | acylphosphatase 1, erythrocyte (common) type | AI325944 | * |
| ADCYAP1R1 | adenylate cyclase activating polypeptide 1 (pituitary) receptor type I | AI846590 | * |
| ADH1C (includes EG:126) | alcohol dehydrogenase 1C (class I), gamma polypeptide | AI850278 | * |
| ADH5 | alcohol dehydrogenase 5 (class III), chi polypeptide | AI848692 | * |
| ADHFE1 | alcohol dehydrogenase, iron containing, 1 | NM_175236 | -8.661 |
| ADIPOR1 | adiponectin receptor 1 | NM_028320 | -4.706 |
| ADNP | activity-dependent neuroprotector | NM_009628 | -3.742 |
| ADSL | adenylosuccinate lyase | NM_009634 | -3.957 |
| AGA | aspartylglucosaminidase | NM_001005847 | -4.651 |
| AHCY | S-adenosylhomocysteine hydrolase | NM_016661 | -5.074 |
| AIFM1 | apoptosis-inducing factor, mitochondrion-associated, 1 | NM_012019 | -5.597 |
| AK1 | adenylate kinase 1 | AI853614 | * |
| AK3L1 | adenylate kinase 3-like 1 | NM_009647 | -5.502 |
| ALDH7A1 | aldehyde dehydrogenase 7 family, member A1 | NM_138600 | -4.3 |
| AMPD1 | adenosine monophosphate deaminase 1 (isoform M) | NM_001033303 | -3.136 |
| AMZ2 | archaemetzincins-2 | AI838636 | * |
| ANGPTL1 | angiopoietin-like 1 | NM_028333 | -3.462 |
| AP3M1 | adaptor-related protein complex 3, mu 1 subunit | NM_018829 | -4.261 |
| AQP4 | aquaporin 4 | NM_009700 | -11.034 |
| ARAF | v-raf murine sarcoma 3611 viral oncogene homolog | AI849825 | * |
| ARFGEF1 | ADP-ribosylation factor guanine nucleotide-exchange factor 1(brefeldin A-inhibited) | XM_975420 | -4.364 |
| ARHGAP26 | Rho GTPase activating protein 26 | NM_175164 | -3.295 |
| ARHGEF3 | Rho guanine nucleotide exchange factor (GEF) 3 | NM_027871 | -3.369 |
| ARL1 | ADP-ribosylation factor-like 1 | NM_025859 | -4.204 |
| ARL4A | ADP-ribosylation factor-like 4A | NM_007487 | -3.929 |
| ARL6IP2 | ADP-ribosylation factor-like 6 interacting protein 2 | NM_019717 | -4.624 |
| ARSG | arylsulfatase G | AI850453 | * |
| ASCC1 | activating signal cointegrator 1 complex subunit 1 | NM_026937 | -4.682 |
| ASF1A | ASF1 anti-silencing function 1 homolog A (S. cerevisiae) | NM_025541 | -3.494 |
| ASPH | aspartate beta-hydroxylase | AI451660 | * |
| ATP2B1 | ATPase, Ca++ transporting, plasma membrane 1 | NM_026482 | -4.487 |
| ATP5B | ATP synthase, H+ transporting, mitochondrial F1 complex, beta polypeptide | NM_016774 | -7.506 |
| ATP5F1 | ATP synthase, H+ transporting, mitochondrial F0 complex, subunit B1 | BC049640 | -4.881 |
| BACE2 | beta-site APP-cleaving enzyme 2 | NM_019517 | -3.778 |
| BANF1 | barrier to autointegration factor 1 | AI850262 | * |
| BAT1 | HLA-B associated transcript 1 | NM_019693 | -3.41 |
| BCAS3 | breast carcinoma amplified sequence 3 | NM_138681 | -4.141 |
| BCKDHA | branched chain keto acid dehydrogenase E1, alpha polypeptide | NM_007533 | -3.648 |
| BLCAP | bladder cancer associated protein | NM_016916 | -4.296 |
| BMI1 | BMI1 polycomb ring finger oncogene | NM_007552 | -6.237 |
| BMP5 | bone morphogenetic protein 5 | NM_007555 | -3.725 |
| BPGM | 2,3-bisphosphoglycerate mutase | NM_007563 | -6.256 |
| BPHL | biphenyl hydrolase-like (serine hydrolase; breast epithelial mucin-associated antigen) | AI846601 | * |
| BRCC3 | BRCA1/BRCA2-containing complex, subunit 3 | NM_145956 | -3.231 |
| BSG | basigin (Ok blood group) | NM_009768 | -3.396 |
| BZW2 | basic leucine zipper and W2 domains 2 | NM_025840 | -4.99 |
| C12ORF5 | chromosome 12 open reading frame 5 | NM_177003 | -3.304 |
| C1D | nuclear DNA-binding protein | NM_020558 | -4.045 |
| C1ORF19 | chromosome 1 open reading frame 19 | AI852014 | * |
| C1ORF96 | chromosome 1 open reading frame 96 | AI447276 | * |
| C20ORF24 | chromosome 20 open reading frame 24 | AI846102 | * |
| C21ORF33 | chromosome 21 open reading frame 33 | NM_138601 | -5.357 |
| C21ORF45 | chromosome 21 open reading frame 45 | AI846439 | * |
| C22ORF9 | chromosome 22 open reading frame 9 | AI846126 | * |
| C5ORF13 | chromosome 5 open reading frame 13 | NM_053078 | -8.415 |
| CA3 | carbonic anhydrase III, muscle specific | NM_007606 | -3.462 |
| CABP1 | calcium binding protein 1 | AI848214 | * |
| CACNA1S | calcium channel, voltage-dependent, L type, alpha 1S subunit | L06234 | -8.872 |
| CACNA2D1 | calcium channel, voltage-dependent, alpha 2/delta subunit 1 | NM_009784 | -4.337 |
| CALM1 | calmodulin 1 (phosphorylase kinase, delta) | NM_009790 | -6.872 |
| CALM2 | calmodulin 2 (phosphorylase kinase, delta) | AI835910 | * |
| CALM3 | calmodulin 3 (phosphorylase kinase, delta) | AI844021 | * |
| CAMK1 | calcium/calmodulin-dependent protein kinase I | AI844640 | * |
| CAMK2G | calcium/calmodulin-dependent protein kinase (CaM kinase) II gamma | AI426269 | * |
| CAPZA2 | capping protein (actin filament) muscle Z-line, alpha 2 | NM_007604 | -3.602 |
| CASKIN1 | CASK interacting protein 1 | AI845923 | * |
| CAV1 | caveolin 1, caveolae protein, 22kDa | NM_007616 | -6.248 |
| CAV2 | caveolin 2 | NM_016900 | -3.596 |
| CBFB | core-binding factor, beta subunit | NM_022309 | -5.482 |
| CCL21 | chemokine (C-C motif) ligand 21 | NM_023052 | -7.058 |
| CCNE2 (includes EG:9134) | cyclin E2 | NM_009830 | -9.768 |
| CCNH | cyclin H | NM_023243 | -3.632 |
| CCNI | cyclin I | AI851548 | * |
| CCT2 | chaperonin containing TCP1, subunit 2 (beta) | NM_007636 | -4.596 |
| CCT6A | chaperonin containing TCP1, subunit 6A (zeta 1) | NM_009838 | -4.778 |
| CD34 | CD34 molecule | AI847784 | * |
| CDC16 | cell division cycle 16 homolog (S. cerevisiae) | NM_027276 | -6.593 |
| CDC25B | cell division cycle 25 homolog B (S. pombe) | AI849132 | * |
| CDH4 | cadherin 4, type 1, R-cadherin (retinal) | NM_009867 | -5.103 |
| CDKL2 | cyclin-dependent kinase-like 2 (CDC2-related kinase) | AI847045 | * |
| CHEK2 | CHK2 checkpoint homolog (S. pombe) | NM_016681 | -3.254 |
| CLCN3 | chloride channel 3 | NM_007711 | -3.503 |
| CLDN22 | claudin 22 | AK008821 | -5.704 |
| CLDN5 | claudin 5 (transmembrane protein deleted in velocardiofacial syndrome) | AI854493 | * |
| CLEC4G | C-type lectin superfamily 4, member G | AI449163 | * |
| CLEC4M | C-type lectin domain family 4, member M | NM_130904 | -4.409 |
| CLIC4 | chloride intracellular channel 4 | AI448908 | * |
| CLK1 | CDC-like kinase 1 | AI850226 | * |
| CMYA5 (includes EG:76469) | cardiomyopathy associated 5 | AJ575748 | -4.536 |
| CNKSR3 | CNKSR family member 3 | NM_172546 | -4.596 |
| CNOT1 | CCR4-NOT transcription complex, subunit 1 | BC018281 | -3.656 |
| CNTNAP2 | contactin associated protein-like 2 | NM_025771 | -6.749 |
| COL4A3BP | collagen, type IV, alpha 3 (Goodpasture antigen) binding protein | AI854393 | * |
| COPB1 | coatomer protein complex, subunit beta 1 | AI840667 | * |
| COX6B2 | cytochrome c oxidase subunit VIb polypeptide 2 (testis) | AI448487 | * |
| CPA3 | carboxypeptidase A3 (mast cell) | NM_007753 | -8.177 |
| CR1 | complement component (3b/4b) receptor 1 (Knops blood group) | NM_013499 | -5.967 |
| CROT | carnitine O-octanoyltransferase | NM_023733 | -4.158 |
| CRSP7 | cofactor required for Sp1 transcriptional activation, subunit 7, 70kDa | NM_027485 | -3.712 |
| CRY1 | cryptochrome 1 (photolyase-like) | NM_007771 | -3.455 |
| CSDE1 | cold shock domain containing E1, RNA-binding | NM_144901 | -4.395 |
| CSE1L | CSE1 chromosome segregation 1-like (yeast) | NM_023565 | -4.37 |
| CSRP3 | cysteine and glycine-rich protein 3 (cardiac LIM protein) | AI385592 | * |
| CSTF1 | cleavage stimulation factor, 3' pre-RNA, subunit 1, 50kDa | AI854108 | * |
| CSTF3 | cleavage stimulation factor, 3' pre-RNA, subunit 3, 77kDa | AI447983 | * |
| CTGF | connective tissue growth factor | AI851778 | * |
| CTNNA3 | catenin (cadherin-associated protein), alpha 3 | NM_177612 | -3.035 |
| CUGBP2 | CUG triplet repeat, RNA binding protein 2 | NM_010160 | -3.446 |
| CUL3 | cullin 3 | AI840051 | * |
| CUZD1 | CUB and zona pellucida-like domains 1 | NM_008411 | -4.426 |
| CXORF40A | chromosome X open reading frame 40A | AI848199 | * |
| CYB5R1 | cytochrome b5 reductase 1 | AI839690 | * |
| CYB5R4 | cytochrome b5 reductase 4 | NM_024195 | -4.394 |
| CYCS | cytochrome c, somatic | AI847355 | * |
| CYFIP2 | cytoplasmic FMR1 interacting protein 2 | NM_133769 | -9.72 |
| CYP39A1 | cytochrome P450, family 39, subfamily A, polypeptide 1 | NM_018887 | -3.835 |
| DAO | D-amino-acid oxidase | AI447515 | * |
| DBT | dihydrolipoamide branched chain transacylase E2 | AI852007 | * |
| DCLK1 | doublecortin-like kinase 1 | NM_019978 | -6.106 |
| DDIT4 | DNA-damage-inducible transcript 4 | AI849939 | * |
| DDX3Y (includes EG:8653) | DEAD (Asp-Glu-Ala-Asp) box polypeptide 3, Y-linked | NM_033077 | -7.893 |
| DKFZP762E1312 | hypothetical protein DKFZp762E1312 | AI847304 | * |
| DKK3 | dickkopf homolog 3 (Xenopus laevis) | NM_015814 | -6.529 |
| DLD | dihydrolipoamide dehydrogenase | NM_007861 | -12.153 |
| DMD | dystrophin (muscular dystrophy, Duchenne and Becker types) | NM_007868 | -4.073 |
| DNAJC12 | DnaJ (Hsp40) homolog, subfamily C, member 12 | AI852445 | * |
| DVL1 | dishevelled, dsh homolog 1 (Drosophila) | AI854114 | * |
| DYNLL2 | dynein, light chain, LC8-type 2 | AI850252 | * |
| DYNLT3 | dynein, light chain, Tctex-type 3 | NM_025975 | -4.524 |
| E2F6 | E2F transcription factor 6 | AI851823 | * |
| EDG1 | endothelial differentiation, sphingolipid G-protein-coupled receptor, 1 | NM_007901 | -4.526 |
| EEF2 | eukaryotic translation elongation factor 2 | NM_007907 | -3.851 |
| EGLN1 | egl nine homolog 1 (C. elegans) | NM_053207 | -4.293 |
| EIF1AX | eukaryotic translation initiation factor 1A, X-linked | NM_025437 | -4.5 |
| EIF3S6 | eukaryotic translation initiation factor 3, subunit 6 48kDa | NM_008388 | -6.68 |
| EIF4A2 | eukaryotic translation initiation factor 4A, isoform 2 | NM_013506 | -9.467 |
| EIF4G2 (includes EG:1982) | eukaryotic translation initiation factor 4 gamma, 2 | NM_013507 | -3.736 |
| EPB41 | erythrocyte membrane protein band 4.1 (elliptocytosis 1, RH-linked) | NM_183428 | -3.084 |
| EPHA7 | EPH receptor A7 | NM_010141 | -4.067 |
| ESR1 | estrogen receptor 1 | NM_007956 | -4.099 |
| ETF1 | eukaryotic translation termination factor 1 | AK139933 | -3.677 |
| EXOSC1 | exosome component 1 | AI848959 | * |
| FAIM | Fas apoptotic inhibitory molecule | AI843459 | * |
| FARSB | phenylalanyl-tRNA synthetase, beta subunit | NM_011811 | -3.236 |
| FBP2 | fructose-1,6-bisphosphatase 2 | NM_007994 | -3.706 |
| FBXL5 | F-box and leucine-rich repeat protein 5 | NM_178729 | -5.196 |
| FECH | ferrochelatase (protoporphyria) | NM_007998 | -3.302 |
| FEM1B | fem-1 homolog b (C. elegans) | AI852273 | * |
| FKBP4 | FK506 binding protein 4, 59kDa | AI848455 | * |
| FKBP8 | FK506 binding protein 8, 38kDa | AI838222 | * |
| FMOD | fibromodulin | NM_021355 | -3.805 |
| FN3K | fructosamine 3 kinase | AI852370 | * |
| FOSB | FBJ murine osteosarcoma viral oncogene homolog B | AI846927 | * |
| FOXO1A | forkhead box O1A | NM_019739 | -3.871 |
| FSTL1 | follistatin-like 1 | AI853088 | * |
| FURIN | furin (paired basic amino acid cleaving enzyme) | AI844976 | * |
| GART | phosphoribosylglycinamide formyltransferase, phosphoribosylglycinamide synthetase, phosphoribosylaminoimidazole synthetase | AI852195 | * |
| GDF8 | growth differentiation factor 8 | NM_010834 | -3.15 |
| GDI2 | GDP dissociation inhibitor 2 | NM_008112 | -3.656 |
| GFRA1 | GDNF family receptor alpha 1 | NM_010279 | -3.538 |
| GLMN | glomulin, FKBP associated protein | NM_133248 | -6.133 |
| GLUL | glutamate-ammonia ligase (glutamine synthetase) | AI848786 | * |
| GNG2 | guanine nucleotide binding protein (G protein), gamma 2 | NM_010315 | -5.021 |
| GNPNAT1 | glucosamine-phosphate N-acetyltransferase 1 | AI852572 | * |
| GOLPH3 | golgi phosphoprotein 3 (coat-protein) | NM_025673 | -3.568 |
| GPC3 | glypican 3 | NM_016697 | -4.55 |
| GPD2 | glycerol-3-phosphate dehydrogenase 2 (mitochondrial) | AK079336 | -3.364 |
| GREM2 | gremlin 2, cysteine knot superfamily, homolog (Xenopus laevis) | NM_011825 | -4.144 |
| GRIK5 | glutamate receptor, ionotropic, kainate 5 | AI840085 | * |
| GRM5 | glutamate receptor, metabotropic 5 | AI850523 | * |
| GRP | gastrin-releasing peptide | NM_175012 | -5.519 |
| GSTK1 | glutathione S-transferase kappa 1 | AI323948 | * |
| GSTM1 | glutathione S-transferase M1 | NM_008183 | -3.535 |
| GSTM2 | glutathione S-transferase M2 (muscle) | AI850208 | * |
| GTF2A2 | general transcription factor IIA, 2, 12kDa | NM_001039519 | -3.051 |
| GTF2F1 | general transcription factor IIF, polypeptide 1, 74kDa | AI853340 | * |
| H19 | H19, imprinted maternally expressed untranslated mRNA | AI838614 | * |
| H3F3A | H3 histone, family 3A | NM_008210 | -4.376 |
| HAT1 | histone acetyltransferase 1 | NM_026115 | -4.42 |
| HECTD1 | HECT domain containing 1 | AI325974 | * |
| HERC2 | hect domain and RLD 2 | AI845558 | * |
| HFE | hemochromatosis | AI850020 | * |
| HIGD1A | HIG1 domain family, member 1A | NM_019814 | -3.81 |
| HIRA | HIR histone cell cycle regulation defective homolog A (S. cerevisiae) | NM_010435 | -3.681 |
| HIST1H3F | histone cluster 1, H3f | AI853124 | * |
| HMGB1 | high-mobility group box 1 | NM_010439 | -4.399 |
| HNMT | histamine N-methyltransferase | NM_080462 | -3.82 |
| HNRPK | heterogeneous nuclear ribonucleoprotein K | NM_025279 | -4.12 |
| HOMER2 | homer homolog 2 (Drosophila) | NM_011983 | -4.47 |
| HOP | homeodomain-only protein | AI848177 | * |
| HOXD10 | homeobox D10 | NM_013554 | -3.331 |
| HRSP12 | heat-responsive protein 12 | AI850467 | * |
| HS3ST5 | heparan sulfate (glucosamine) 3-O-sulfotransferase 5 | AK031910 | -5.898 |
| HSMPP8 | M-phase phosphoprotein, mpp8 | AI660999 | * |
| HTRA2 | HtrA serine peptidase 2 | AI847573 | * |
| HUWE1 | HECT, UBA and WWE domain containing 1 | AI326850 | * |
| IARS (includes EG:3376) | isoleucyl-tRNA synthetase | NM_172015 | -3.389 |
| IDH2 | isocitrate dehydrogenase 2 (NADP+), mitochondrial | NM_173011 | -4.175 |
| IFNAR1 | interferon (alpha, beta and omega) receptor 1 | AI447587 | * |
| IL15 | interleukin 15 | NM_008357 | -5.586 |
| INMT | indolethylamine N-methyltransferase | NM_009349 | -3.6 |
| IPO11 | importin 11 | AI850518 | * |
| IRG1 | immunoresponsive gene 1 | AI323667 | * |
| IRS1 | insulin receptor substrate 1 | AI839755 | * |
| ISG20L1 | interferon stimulated exonuclease gene 20kDa-like 1 | NM_026531 | -3.416 |
| ITGB4BP | integrin beta 4 binding protein | NM_010579 | -3.433 |
| ITM2A | integral membrane protein 2A | NM_008409 | -6.202 |
| IVNS1ABP | influenza virus NS1A binding protein | NM_001039511 | -4.917 |
| JAM2 | junctional adhesion molecule 2 | NM_023844 | -3.972 |
| JTV1 | JTV1 gene | NM_146165 | -6.564 |
| KCNAB1 | potassium voltage-gated channel, shaker-related subfamily, beta member 1 | NM_010597 | -4.236 |
| KCNC1 | potassium voltage-gated channel, Shaw-related subfamily, member 1 | AI844638 | * |
| KCNG4 | potassium voltage-gated channel, subfamily G, member 4 | NM_025734 | -5.599 |
| KEAP1 | kelch-like ECH-associated protein 1 | AI854035 | * |
| KIAA0368 | KIAA0368 | AK030369 | -3.999 |
| KIAA1274 | KIAA1274 | AI852379 | * |
| KIAA1434 | hypothetical protein KIAA1434 | NM_027096 | -5.72 |
| KIF22 | kinesin family member 22 | NM_145588 | -5.506 |
| KLC2 | kinesin light chain 2 | AI414599 | * |
| KLF15 | Kruppel-like factor 15 | NM_023184 | -4.242 |
| KLK8 | kallikrein-related peptidase 8 | NM_008940 | -3.419 |
| LAP3 | leucine aminopeptidase 3 | AI839225 | * |
| LCK | lymphocyte-specific protein tyrosine kinase | AI573454 | * |
| LGTN | ligatin | NM_010709 | -6.043 |
| LLGL1 | lethal giant larvae homolog 1 (Drosophila) | AI854182 | * |
| LRIG1 | leucine-rich repeats and immunoglobulin-like domains 1 | AI853052 | * |
| LYPLA1 | lysophospholipase I | NM_008866 | -6.066 |
| LYPLA2 | lysophospholipase II | AI853141 | * |
| MAFA | v-maf musculoaponeurotic fibrosarcoma oncogene homolog A (avian) | NM_194350 | -5.051 |
| MAG (includes EG:4099) | myelin associated glycoprotein | AI853430 | * |
| MAGEE1 | melanoma antigen family E, 1 | AI413449 | * |
| MAP2K5 | mitogen-activated protein kinase kinase 5 | NM_011840 | -3.736 |
| MAT2B | methionine adenosyltransferase II, beta | NM_134017 | -4.848 |
| MBD4 | methyl-CpG binding domain protein 4 | AI465519 | * |
| MBNL2 | muscleblind-like 2 (Drosophila) | NM_175341 | -6.8 |
| MCCC1 | methylcrotonoyl-Coenzyme A carboxylase 1 (alpha) | NM_023644 | -3.784 |
| MCFD2 | multiple coagulation factor deficiency 2 | NM_139295 | -5.041 |
| MCL1 | myeloid cell leukemia sequence 1 (BCL2-related) | AI838732 | * |
| MCPT4 (includes EG:17227) | mast cell protease 4 | NM_010779 | -4.049 |
| MDC1 | mediator of DNA damage checkpoint 1 | AI447990 | * |
| MEF2C | myocyte enhancer factor 2C | AI852387 | * |
| MEIS2 | Meis homeobox 2 | AI849251 | * |
| MGC4093 | hypothetical protein MGC4093 | AI846557 | * |
| MGRN1 | mahogunin, ring finger 1 | AI661152 | * |
| MIA3 | melanoma inhibitory activity family, member 3 | AI385644 | * |
| MMP13 | matrix metallopeptidase 13 (collagenase 3) | AI841659 | * |
| MMRN1 | multimerin 1 | XM_284198 | -4.566 |
| MTHFD1 | methylenetetrahydrofolate dehydrogenase (NADP+ dependent) 1, methenyltetrahydrofolate cyclohydrolase, formyltetrahydrofolate synthetase | NM_138745 | -4.235 |
| MTM1 | myotubularin 1 | NM_019926 | -4.024 |
| MTMR14 | myotubularin related protein 14 | NM_026849 | -7.193 |
| MUTED | muted homolog (mouse) | NM_139063 | -4.072 |
| MYBBP1A | MYB binding protein (P160) 1a | AI414970 | * |
| MYH2 | myosin, heavy chain 2, skeletal muscle, adult | NM_001039545 | -5.268 |
| MYH6 | myosin, heavy chain 6, cardiac muscle, alpha (cardiomyopathy, hypertrophic 1) | NM_010856 | -6.412 |
| MYH7 | myosin, heavy chain 7, cardiac muscle, beta | NM_080728 | -5.947 |
| MYL2 | myosin, light chain 2, regulatory, cardiac, slow | NM_010861 | -11.103 |
| MYL3 | myosin, light chain 3, alkali; ventricular, skeletal, slow | NM_010859 | -3.971 |
| NAP1L4 | nucleosome assembly protein 1-like 4 | NM_008672 | -4.06 |
| NARF | nuclear prelamin A recognition factor | NM_026272 | -3.196 |
| NCKAP1 | NCK-associated protein 1 | AI852043 | * |
| NDEL1 | nudE nuclear distribution gene E homolog (A. nidulans)-like 1 | NM_023668 | -3.649 |
| NDUFAF1 | NADH dehydrogenase (ubiquinone) 1 alpha subcomplex, assembly factor 1 | AI850186 | * |
| NDUFB5 | NADH dehydrogenase (ubiquinone) 1 beta subcomplex, 5, 16kDa | AI844884 | * |
| NEU1 | sialidase 1 (lysosomal sialidase) | AI848918 | * |
| NIP7 | nuclear import 7 homolog (S. cerevisiae) | AI853299 | * |
| NNT | nicotinamide nucleotide transhydrogenase | NM_008710 | -4.272 |
| NOV | nephroblastoma overexpressed gene | AI838492 | * |
| NPY1R | neuropeptide Y receptor Y1 | NM_010934 | -5.67 |
| NR3C1 | nuclear receptor subfamily 3, group C, member 1 (glucocorticoid receptor) | NM_008173 | -7.087 |
| NRBP1 | nuclear receptor binding protein 1 | NM_147201 | -4.905 |
| NRGN | neurogranin (protein kinase C substrate, RC3) | AI326818 | * |
| NUDT4 | nudix (nucleoside diphosphate linked moiety X)-type motif 4 | NM_027722 | -3.759 |
| NUP205 | nucleoporin 205kDa | XM_133073 | -3.98 |
| NUP210 | nucleoporin 210kDa | AI854072 | * |
| NUP37 | nucleoporin 37kDa | NM_028334 | -3.415 |
| NUP43 | nucleoporin 43kDa | NM_145706 | -4.395 |
| NXF1 | nuclear RNA export factor 1 | NM_016813 | -3.894 |
| OMA1 | OMA1 homolog, zinc metallopeptidase (S. cerevisiae) | NM_025909 | -4.385 |
| ORC4L | origin recognition complex, subunit 4-like (yeast) | NM_011958 | -5.053 |
| OSTN | osteocrin | NM_198112 | -5.391 |
| P2RY1 | purinergic receptor P2Y, G-protein coupled, 1 | NM_008772 | -4.302 |
| PABPC4 | poly(A) binding protein, cytoplasmic 4 (inducible form) | NM_148917 | -4.147 |
| PAICS | phosphoribosylaminoimidazole carboxylase, phosphoribosylaminoimidazole succinocarboxamide synthetase | NM_025939 | -6.768 |
| PAIP1 | poly(A) binding protein interacting protein 1 | AK142913 | -5.054 |
| PAX6 | paired box gene 6 (aniridia, keratitis) | AI852835 | * |
| PBEF1 | pre-B-cell colony enhancing factor 1 | NM_021524 | -4.137 |
| PCTK3 | PCTAIRE protein kinase 3 | AI323675 | * |
| PDLIM5 | PDZ and LIM domain 5 | NM_019809 | -4.986 |
| PDPK1 | 3-phosphoinositide dependent protein kinase-1 | NM_011062 | -4.126 |
| PECAM1 | platelet/endothelial cell adhesion molecule (CD31 antigen) | NM_008816 | -3.543 |
| PEO1 | progressive external ophthalmoplegia 1 | NM_153796 | -4.278 |
| PFN2 | profilin 2 | NM_019410 | -5.999 |
| PHKB | phosphorylase kinase, beta | NM_199446 | -3.778 |
| PHLDB1 | pleckstrin homology-like domain, family B, member 1 | AI854506 | * |
| PIGY | phosphatidylinositol glycan anchor biosynthesis, class Y | AI847956 | * |
| PITPNC1 | phosphatidylinositol transfer protein, cytoplasmic 1 | NM_145823 | -4.652 |
| PKD2 (includes EG:5311) | polycystic kidney disease 2 (autosomal dominant) | AI414001 | * |
| PKIA | protein kinase (cAMP-dependent, catalytic) inhibitor alpha | NM_008862 | -5.019 |
| PLEKHA1 | pleckstrin homology domain containing, family A (phosphoinositide binding specific) member 1 | AI849556 | * |
| PLEKHB2 | pleckstrin homology domain containing, family B (evectins) member 2 | NM_145516 | -4.149 |
| PLP1 | proteolipid protein 1 (Pelizaeus-Merzbacher disease, spastic paraplegia 2, uncomplicated) | AI848420 | * |
| PLXNA2 | plexin A2 | AI844350 | * |
| POLR2D | polymerase (RNA) II (DNA directed) polypeptide D | AI844606 | * |
| POLR2J | polymerase (RNA) II (DNA directed) polypeptide J, 13.3kDa | AI846544 | * |
| POLR3D | polymerase (RNA) III (DNA directed) polypeptide D, 44kDa | AI844711 | * |
| PPBP | pro-platelet basic protein (chemokine (C-X-C motif) ligand 7) | AI854500 | * |
| PPP1R1A | protein phosphatase 1, regulatory (inhibitor) subunit 1A | NM_021391 | -8.703 |
| PPP1R3C | protein phosphatase 1, regulatory (inhibitor) subunit 3C | NM_016854 | -17.773 |
| PPP1R7 | protein phosphatase 1, regulatory (inhibitor) subunit 7 | NM_023200 | -3.301 |
| PPP2CB | protein phosphatase 2 (formerly 2A), catalytic subunit, beta isoform | NM_017374 | -4.43 |
| PPP2R2A | protein phosphatase 2 (formerly 2A), regulatory subunit B, alpha isoform | AK010380 | -3.559 |
| PPP3CB | protein phosphatase 3 (formerly 2B), catalytic subunit, beta isoform | NM_008914 | -4.928 |
| PRELP | proline/arginine-rich end leucine-rich repeat protein | AI851932 | * |
| PRKAG1 | protein kinase, AMP-activated, gamma 1 non-catalytic subunit | NM_016781 | -3.71 |
| PRKAR2A | protein kinase, cAMP-dependent, regulatory, type II, alpha | NM_008924 | -5.393 |
| PRKRIR | protein-kinase, interferon-inducible double stranded RNA dependent inhibitor, repressor of (P58 repressor) | NM_028410 | -6.204 |
| PRMT7 | protein arginine methyltransferase 7 | NM_145404 | -3.475 |
| PRPF4B | PRP4 pre-mRNA processing factor 4 homolog B (yeast) | NM_013830 | -3.435 |
| PRPS1 | phosphoribosyl pyrophosphate synthetase 1 | NM_021463 | -5.003 |
| PSMB9 | proteasome (prosome, macropain) subunit, beta type, 9 (large multifunctional peptidase 2) | AI323618 | * |
| PSMD12 | proteasome (prosome, macropain) 26S subunit, non-ATPase, 12 | NM_025894 | -4.973 |
| PSMD13 | proteasome (prosome, macropain) 26S subunit, non-ATPase, 13 | NM_011875 | -3.652 |
| PSMD14 | proteasome (prosome, macropain) 26S subunit, non-ATPase, 14 | NM_021526 | -3.631 |
| PSMD6 | proteasome (prosome, macropain) 26S subunit, non-ATPase, 6 | AI847475 | * |
| PTGES3 | prostaglandin E synthase 3 (cytosolic) | AI845883 | * |
| PTGFR | prostaglandin F receptor (FP) | NM_008966 | -4.001 |
| PXMP3 | peroxisomal membrane protein 3, 35kDa (Zellweger syndrome) | NM_008994 | -6.116 |
| PYCR2 | pyrroline-5-carboxylate reductase family, member 2 | AI847678 | * |
| RAB18 | RAB18, member RAS oncogene family | NM_011225 | -5.307 |
| RAB7A | RAB7A, member RAS oncogene family | NM_009005 | -3.604 |
| RAD21 | RAD21 homolog (S. pombe) | NM_009009 | -4.614 |
| RBL2 | retinoblastoma-like 2 (p130) | NM_011250 | -6.259 |
| RBP7 | retinol binding protein 7, cellular | NM_022020 | -4.497 |
| RCHY1 | ring finger and CHY zinc finger domain containing 1 | NM_026557 | -3.582 |
| RETN | resistin | NM_022984 | -3.281 |
| REXO2 | REX2, RNA exonuclease 2 homolog (S. cerevisiae) | AI839882 | * |
| RFK | riboflavin kinase | NM_019437 | -3.546 |
| RFXAP | regulatory factor X-associated protein | AI849973 | * |
| RGS5 | regulator of G-protein signalling 5 | AK028765 | -6.132 |
| RHOG | ras homolog gene family, member G (rho G) | AI834967 | * |
| RNF141 | ring finger protein 141 | NM_025999 | -3.51 |
| RNF6 | ring finger protein (C3H2C3 type) 6 | NM_028774 | -5.454 |
| RNF8 | ring finger protein 8 | NM_021419 | -4.105 |
| RPL22 | ribosomal protein L22 | AI447249 | * |
| RPL36 | ribosomal protein L36 | AI464570 | * |
| RPL4 | ribosomal protein L4 | NM_024212 | -4.49 |
| RPP40 | ribonuclease P 40kDa subunit | NM_145938 | -3.064 |
| RPS6KC1 | ribosomal protein S6 kinase, 52kDa, polypeptide 1 | NM_178775 | -3.565 |
| RRAGB | Ras-related GTP binding B | NM_001004154 | -3.576 |
| RSL1D1 | ribosomal L1 domain containing 1 | NM_025546 | -3.256 |
| RTN4 | reticulon 4 | NM_194054 | -4.51 |
| RUVBL1 | RuvB-like 1 (E. coli) | NM_019685 | -3.684 |
| SAE1 | SUMO1 activating enzyme subunit 1 | NM_019748 | -4.283 |
| SAR1B | SAR1 gene homolog B (S. cerevisiae) | NM_025535 | -4.131 |
| SC5DL | sterol-C5-desaturase (ERG3 delta-5-desaturase homolog, S. cerevisiae)-like | AI845765 | * |
| SCN1B | sodium channel, voltage-gated, type I, beta | NM_011322 | -3.64 |
| SDC2 | syndecan 2 | AI852485 | * |
| SDHA | succinate dehydrogenase complex, subunit A, flavoprotein (Fp) | NM_023281 | -4.551 |
| SDHB | succinate dehydrogenase complex, subunit B, iron sulfur (Ip) | NM_023374 | -4.286 |
| SEC13 | SEC13 homolog (S. cerevisiae) | NM_024206 | -4.83 |
| SEC23A | Sec23 homolog A (S. cerevisiae) | NM_009147 | -5.39 |
| SEC24A | SEC24 related gene family, member A (S. cerevisiae) | AK090341 | -3.4 |
| SERPIND1 | serpin peptidase inhibitor, clade D (heparin cofactor), member 1 | AI850820 | * |
| SESN1 | sestrin 1 | NM_001013370 | -4.862 |
| SFXN1 | sideroflexin 1 | AI853409 | * |
| SGCB | sarcoglycan, beta (43kDa dystrophin-associated glycoprotein) | AI844132 | * |
| SH3GLB1 | SH3-domain GRB2-like endophilin B1 | NM_019464 | -3.375 |
| SH3KBP1 | SH3-domain kinase binding protein 1 | NM_021389 | -4.825 |
| SHMT2 | serine hydroxymethyltransferase 2 (mitochondrial) | AI848189 | * |
| SIAHBP1 | fuse-binding protein-interacting repressor | AI837793 | * |
| SLC16A10 | solute carrier family 16, member 10 (aromatic amino acid transporter) | NM_028247 | -3.412 |
| SLC25A12 | solute carrier family 25 (mitochondrial carrier, Aralar), member 12 | AK140681 | -10.979 |
| SLC25A3 | solute carrier family 25 (mitochondrial carrier; phosphate carrier), member 3 | AK028313 | -6.485 |
| SLC30A9 | solute carrier family 30 (zinc transporter), member 9 | NM_178651 | -3.85 |
| SMOC2 | SPARC related modular calcium binding 2 | NM_022315 | -11.361 |
| SMPDL3A | sphingomyelin phosphodiesterase, acid-like 3A | AI849987 | * |
| SNAPAP | SNAP-associated protein | AI852926 | * |
| SNURF | SNRPN upstream reading frame | NM_033174 | -3.767 |
| SOD2 | superoxide dismutase 2, mitochondrial | AK035485 | -3.698 |
| SORBS2 | sorbin and SH3 domain containing 2 | AI851858 | * |
| SPARC | secreted protein, acidic, cysteine-rich (osteonectin) | AI840197 | * |
| SPON1 | spondin 1, extracellular matrix protein | NM_145584 | -6.206 |
| SRA1 (includes EG:10011) | steroid receptor RNA activator 1 | AI837240 | * |
| SRPR | signal recognition particle receptor ('docking protein') | NM_026130 | -4.787 |
| ST7 | suppression of tumorigenicity 7 | NM_022332 | -3.452 |
| STAT1 | signal transducer and activator of transcription 1, 91kDa | AI449540 | * |
| SUB1 | SUB1 homolog (S. cerevisiae) | AI844608 | * |
| SUCLG2 | succinate-CoA ligase, GDP-forming, beta subunit | NM_011507 | -6.859 |
| SURF5 | surfeit 5 | NM_011513 | -4.201 |
| SUV420H2 | suppressor of variegation 4-20 homolog 2 (Drosophila) | AI661477 | * |
| SYBL1 | synaptobrevin-like 1 | NM_011515 | -3.512 |
| SYNPO2 | synaptopodin 2 | XM_619958 | -4.887 |
| SYPL1 | synaptophysin-like 1 | NM_013635 | -3.735 |
| TACC1 | transforming, acidic coiled-coil containing protein 1 | AI447878 | * |
| TAF12 | TAF12 RNA polymerase II, TATA box binding protein (TBP)-associated factor, 20kDa | AI853687 | * |
| TAF9B | TAF9B RNA polymerase II, TATA box binding protein (TBP)-associated factor, 31kDa | AI850390 | * |
| TANK | TRAF family member-associated NFKB activator | NM_011529 | -5.374 |
| TARSL2 | threonyl-tRNA synthetase-like 2 | NM_172310 | -5.283 |
| TBCE | tubulin folding cofactor E | NM_178337 | -3.41 |
| TDRD3 | tudor domain containing 3 | AK078326 | -5.16 |
| TFEB | transcription factor EB | AI853751 | * |
| TFPI | tissue factor pathway inhibitor (lipoprotein-associated coagulation inhibitor) | NM_011576 | -4.651 |
| TGTP | T-cell specific GTPase | NM_011579 | -6.621 |
| THBD | thrombomodulin | AI385582 | * |
| THBS4 | thrombospondin 4 | NM_011582 | -3.236 |
| TIMP4 | TIMP metallopeptidase inhibitor 4 | NM_080639 | -4.041 |
| TLK2 (includes EG:11011) | tousled-like kinase 2 | AI845045 | * |
| TM2D1 | TM2 domain containing 1 | NM_053157 | -3.746 |
| TMED2 | transmembrane emp24 domain trafficking protein 2 | NM_019770 | -3.83 |
| TMEM126A | transmembrane protein 126A | NM_025460 | -3.084 |
| TMEM49 | transmembrane protein 49 | NM_029478 | -3.716 |
| TNMD | tenomodulin | NM_022322 | -4.976 |
| TOM1 | target of myb1 (chicken) | NM_011622 | -4.061 |
| TRAM1 | translocation associated membrane protein 1 | AI844979 | * |
| TRIM23 | tripartite motif-containing 23 | NM_030731 | -4.444 |
| TRIP11 | thyroid hormone receptor interactor 11 | AI450776 | * |
| TRNT1 | tRNA nucleotidyl transferase, CCA-adding, 1 | AI845098 | * |
| TSC1 | tuberous sclerosis 1 | AI837319 | * |
| TSPAN3 | tetraspanin 3 | NM_019793 | -3.595 |
| TSPAN8 | tetraspanin 8 | NM_146010 | -3.854 |
| TSTA3 | tissue specific transplantation antigen P35B | AI842571 | * |
| TTC28 | tetratricopeptide repeat domain 28 | AI848701 | * |
| TUBA4A | tubulin, alpha 4a | NM_009447 | -4.136 |
| TUBB | tubulin, beta | AI837422 | * |
| UBE2A | ubiquitin-conjugating enzyme E2A (RAD6 homolog) | AI854872 | * |
| UBE2B | ubiquitin-conjugating enzyme E2B (RAD6 homolog) | NM_009458 | -3.286 |
| UBE2D1 | ubiquitin-conjugating enzyme E2D 1 (UBC4/5 homolog, yeast) | NM_145420 | -3.384 |
| UBE2G1 | ubiquitin-conjugating enzyme E2G 1 (UBC7 homolog, yeast) | NM_025985 | -4.39 |
| UBQLN2 | ubiquilin 2 | AI854423 | * |
| ULK2 | unc-51-like kinase 2 (C. elegans) | NM_013881 | -3.453 |
| UQCRC2 | ubiquinol-cytochrome c reductase core protein II | AI840110 | * |
| UQCRFS1 | ubiquinol-cytochrome c reductase, Rieske iron-sulfur polypeptide 1 | AI850420 | * |
| USP1 | ubiquitin specific peptidase 1 | AI848382 | * |
| USP15 | ubiquitin specific peptidase 15 | NM_027604 | -3.967 |
| USP38 | ubiquitin specific peptidase 38 | NM_027554 | -4.384 |
| VAMP3 | vesicle-associated membrane protein 3 (cellubrevin) | NM_009498 | -5.083 |
| VAPB | VAMP (vesicle-associated membrane protein)-associated protein B and C | NM_019806 | -3.379 |
| VBP1 | von Hippel-Lindau binding protein 1 | NM_011692 | -3.522 |
| VPS35 | vacuolar protein sorting 35 homolog (S. cerevisiae) | NM_022997 | -4.801 |
| VTI1B | vesicle transport through interaction with t-SNAREs homolog 1B (yeast) | AI839759 | * |
| VWF | von Willebrand factor | NM_011708 | -3.52 |
| WDR12 | WD repeat domain 12 | NM_021312 | -3.82 |
| WDR36 | WD repeat domain 36 | NM_144863 | -4.368 |
| WDR48 | WD repeat domain 48 | AI852916 | * |
| WDR77 | WD repeat domain 77 | NM_027432 | -5.168 |
| WIF1 | WNT inhibitory factor 1 | NM_011915 | -12.265 |
| WNT4 | wingless-type MMTV integration site family, member 4 | NM_009523 | -3.868 |
| WWP1 | WW domain containing E3 ubiquitin protein ligase 1 | AK033138 | -5.094 |
| XAB1 | XPA binding protein 1, GTPase | AI839342 | * |
| XIST | X (inactive)-specific transcript | L04961 | -86.22 |
| XRCC6 | X-ray repair complementing defective repair in Chinese hamster cells 6 (Ku autoantigen, 70kDa) | AI854574 | * |
| YKT6 | YKT6 v-SNARE homolog (S. cerevisiae) | AI844927 | * |
| YME1L1 | YME1-like 1 (S. cerevisiae) | NM_013771 | -3.458 |
| YY1 | YY1 transcription factor | AI842603 | * |
| ZC3H8 | zinc finger CCCH-type containing 8 | AI448873 | * |
| ZHX1 | zinc fingers and homeoboxes 1 | NM_009572 | -3.784 |
| ZIC1 | Zic family member 1 (odd-paired homolog, Drosophila) | AI848240 | * |
| ZMYND19 | zinc finger, MYND-type containing 19 | NM_026021 | -3.632 |
| ZNF160 | zinc finger protein 160 | AI848426 | * |
| ZNF365 | zinc finger protein 365 | AI839779 | * |

A fold change value is provided for genes identified on the Agilent arrays at 14 days post Dox treatment. A * denotes that the gene was identified on the BMAP arrays and had a fold change greater than -3 for at least one of the time points tested 0, 4, 7, 14, 30 or 60 days post Dox treatment.
